# Trim-Away ubiquitinates and degrades lysine-less and N-terminally acetylated substrates

**DOI:** 10.1038/s41467-023-37504-x

**Published:** 2023-04-15

**Authors:** Leo Kiss, Tyler Rhinesmith, Jakub Luptak, Claire F. Dickson, Jonas Weidenhausen, Shannon Smyly, Ji-Chun Yang, Sarah L. Maslen, Irmgard Sinning, David Neuhaus, Dean Clift, Leo C. James

**Affiliations:** 1 MRC Laboratory of Molecular Biology, Francis Crick Avenue, Cambridge, CB2 0QH UK; 2 EMBL Australia Node in Single Molecule Science and ARC Centre of Excellence in Advanced Molecular Imaging School of Medical Sciences, UNSW Sydney, NSW 2052 Australia; 3 Biochemiezentrum der Universität Heidelberg (BZH), INF328, D-69120 Heidelberg, Germany; 4 Present Address: Max Planck Institute of Biochemistry, Am Klopferspitz 18, 82152 Martinsried, Germany; 5 Present Address: EMBL Heidelberg, Meyerhofstraße 1, 69117 Heidelberg, Germany; 6 Present Address: The Francis Crick Institute, 1 Midland Road, London, NW1 1AT UK

**Keywords:** Ubiquitylation, Ubiquitin ligases, Antimicrobial responses, Enzyme mechanisms

## Abstract

TRIM proteins are the largest family of E3 ligases in mammals. They include the intracellular antibody receptor TRIM21, which is responsible for mediating targeted protein degradation during Trim-Away. Despite their importance, the ubiquitination mechanism of TRIM ligases has remained elusive. Here we show that while Trim-Away activation results in ubiquitination of both ligase and substrate, ligase ubiquitination is not required for substrate degradation. N-terminal TRIM21 RING ubiquitination by the E2 Ube2W can be inhibited by N-terminal acetylation, but this doesn’t prevent substrate ubiquitination nor degradation. Instead, uncoupling ligase and substrate degradation prevents ligase recycling and extends functional persistence in cells. Further, Trim-Away degrades substrates irrespective of whether they contain lysines or are N-terminally acetylated, which may explain the ability of TRIM21 to counteract fast-evolving pathogens and degrade diverse substrates.

## Abstract (web summary)

TRIM21 mediates intracellular antibody immunity and is exploited for targeted protein degradation using Trim-Away technology. Here, the authors dissect the ubiquitination requirements for Trim-Away, providing an explanation for how TRIM21 can target diverse substrates for degradation.

## Introduction

TRIM proteins constitute the largest family of RING E3 ligases in mammals. They include TRIMs that suppress viral infection (TRIM5^1^, TRIM21^2^, TRIM22^3^, TRIM25^4^), activate innate immunity (TRIM32^5^, TRIM56^6^, TRIM65^7^, RIPLET^8^), and repress transcription (TRIM4^9^, TRIM28^10^). Unlike cullin-RING ubiquitin ligases (CRLs) that use a modular system of RINGs, adaptors and scaffolds to create distinct enzymes^11,12^, TRIM ligases contain both substrate-targeting and catalytic domains in one polyprotein. How TRIMs catalyze ubiquitination is incompletely understood, particularly in terms of activation, ubiquitin priming and chain extension. This is due in part to the difficulty linking in vitro activity with cellular function. For instance, many RINGs have been shown to work with E2s in vitro for which there is no data supporting a cellular role.

Current mechanisms of TRIM catalysis have been informed primarily by experiments on the two antiviral proteins TRIM5 and TRIM21. Both proteins are dimers containing a RING, B Box, coiled-coil and PRYSPRY domains. Each RING domain is arranged at opposite ends of the elongated antiparallel coiled-coil^13^ and whilst ubiquitination of monomeric RINGs can be detected in vitro^14^, dimerization is required for full cellular activity^15,16^. Intramolecular RING dimerization would require an extensive conformational rearrangement, involving an extreme bend angle in the coiled-coil, and existing data suggests that TRIM RINGs instead undergo intermolecular dimerization through a mechanism of substrate-induced clustering^15^. In the case of TRIM5, this occurs during binding to the conical capsid of HIV-1^17^: The primarily hexameric capsid induces formation of a hexameric lattice of TRIM5 molecules^18^ anchored to the capsid surface through PRYSPRY domain interactions^17^. The TRIM5 lattice is further stabilized through trimeric contacts formed between the B Box domains at each vertex^19^ and transient RING dimerization^19,20^. TRIM21 also undergoes supramolecular clustering^15^, including on the surface of viral capsids^21^, but is anchored to its substrates by an intermediate antibody molecule^2^: The Fabs of each antibody bind the substrate whilst the Fc is bound by the TRIM21 PRYSPRY^22^. There is no evidence that TRIM21 forms a regular structure or that its B Box mediates oligomerization. Instead, the B Box of TRIM21 is an autoinhibitory domain that supresses RING activity in the non-clustered state by competing for E2–Ub binding^14^. Supramolecular assembly is sufficient for TRIM RING activation. In the case of TRIM21, light-induced clustering of a cryptochrome2-TRIM21 fusion triggered its TRIM21 RING- and proteasome-dependent degradation^15^. Meanwhile, TRIM5 degradation was accelerated in the presence of HIV-1 capsid^23^, and prevented by a single B Box mutation that prevents higher order assembly^24^.

A further important difference between TRIM and CRL ligases is that the former undergoes degradation along with its substrate. This has been shown for TRIM5 during HIV infection^23^ and for TRIM21 with a wide-range of substrates during Trim-Away^25^. Moreover, TRIM21 and its substrates are degraded with matching kinetics suggesting that they are processed together as a complex^25^. In support of TRIM ligase self-degradation, TRIM21 degradation can be triggered by inducing its clustering independently of antibody or substrate binding^15^. Meanwhile, TRIM5 self-degradation can be induced simply by ectopic overexpression^26^, which leads to the formation of large oligomers called ‘cytoplasmic bodies’, likely driven by B Box trimerization^19,24^.

Activation of TRIM RINGs enables E2 recruitment and the catalysis of ubiquitination. Multiple E2s have been reported as partners but only depletion of the N-terminal monoubiquitinating E2 Ube2W or the K63-chain forming heterodimer Ube2N/Ube2V2 has been shown to inhibit the cellular function of TRIM5^27,28^ and TRIM21^29,30^. Moreover, point mutations in TRIM21 that specifically inhibit catalysis with Ube2N resulted in loss of cellular function^15,31^. K63-chain ubiquitination has also been implicated in the function of many other TRIMs, such as TRIM4^32^, TRIM8^33^, TRIM22^34^, TRIM31^35^, TRIM34^36^, TRIM54^37^, TRIM59^38^, TRIM65^7^ and RIPLET^8^. In vitro, both TRIM5 and TRIM21 have been shown to catalyze monoubiquitination of their own N-terminus when incubated with Ube2W, whilst the addition of Ube2N/Ube2V2 drives chain extension to produce an anchored K63 chain^27,29^. This K63-linked autoubiquitination can be detected in cells during substrate engagement^15^ or over-expression and is reversed by Ube2W or Ube2N depletion^27,29^. Substrate-induced RING activation can also be reproduced in vitro, with the addition of IgG Fc promoting the formation of anchored K63-chains on TRIM21^39^. Moreover, in vitro experiments suggest that RING clustering is important not only to generate active RING dimers but also to allow intermolecular RING ubiquitination. A ‘two-plus-one’ model has been demonstrated, in which a RING dimer ubiquitinates the N-terminus of a neighboring RING monomer^20^. For TRIM5, this is supported by in vitro ubiquitination rescue experiments using catalytically dead mutants^20^ and structural data demonstrating a trimeric RING arrangement in assembled TRIM5 lattices^18,19,40^. For TRIM21, RING dimerization was shown to be insufficient for effective substrate-induced ubiquitination, with full activity requiring the recruitment of two RING dimers^39^.

This correspondence between the functional requirement for TRIM clustering and the mechanistic requirement for higher-order catalytic RING topology helps explain how TRIMs are activated and regulated. However, how K63-ubiquitination facilitates proteasomal degradation is less clear. It has been suggested that TRIM-synthesized K63-chains are further modified with branched K48-chains, similar to that reported for UBR5, HUWE1, UBR4 and cIAP1^41–43^. In support of this, both K63 and K48-chains can be detected on overexpressed TRIM21 and are lost concomitantly upon either Ube2W or Ube2N depletion^29^. Importantly, while autoubiquitination explains why TRIM ligases are degraded upon activation, it does not provide a direct mechanism for substrate degradation. Whether substrates are also modified with ubiquitin during their cellular degradation is unknown, despite attempts to detect this^2,17^, and so because of the absence of such data it has been proposed that ligase autoubiquitination alone may drive proteasome recruitment, resulting in degradation of the entire TRIM:substrate complex^2,26,44,45^.

Here we sought to test the requirement for TRIM autoubiquitination in substrate degradation. Using Trim-Away as a model system, we show that whilst N-terminal ubiquitination drives ligase turnover it is not required for substrate degradation. Rather, uncoupling ligase and substrate degradation prolongs ligase lifetime allowing it to persist in cells for longer. We demonstrate, both in vitro and in cells, direct substrate ubiquitination by endogenous TRIM21 and TRIM21 RING-containing Trim-Away constructs and show that this is unaffected by inhibiting N-terminal RING autoubiquitination. Finally, we establish a cellular Trim-Away assay in which all lysines can be removed or mutated to arginine and substrate N-terminal ubiquitination can be blocked. Surprisingly, we find that ubiquitination and degradation does not require an available lysine or N-termini in either the ligase or substrate, which may explain how TRIM21 achieves such broad substrate specificity.

## Results

### Structure of TRIM21 RING in complex with Ube2W

**Fig. 1:**
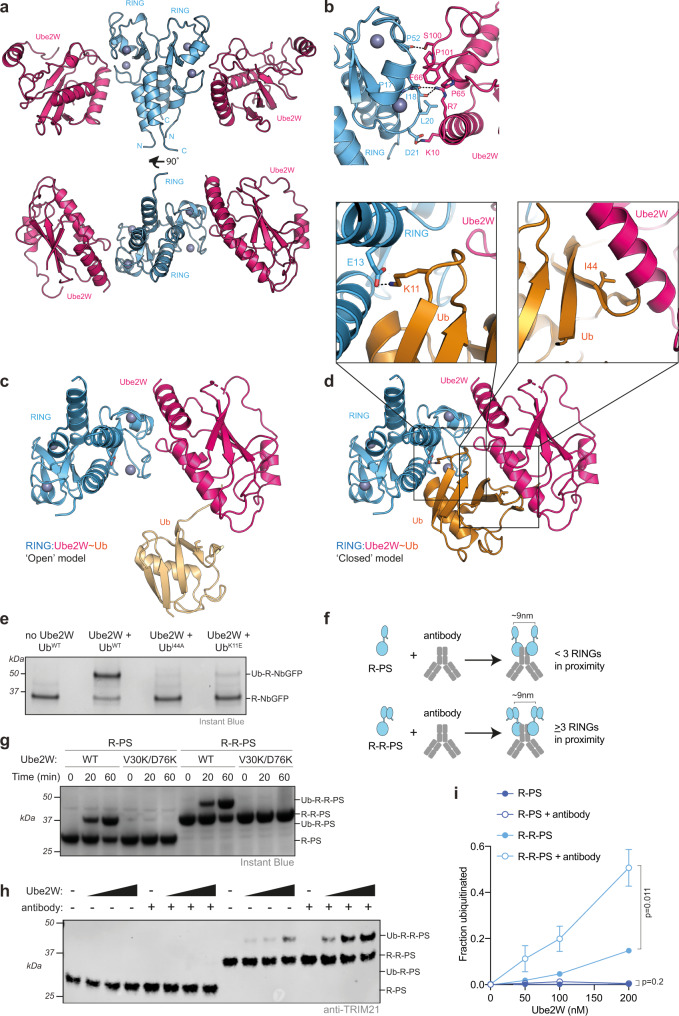
Dimeric Ube2W and RING clustering is required for ligase autoubiquitination. **a** 2.25 Å X-ray structure of TRIM21 RING (blue) in complex with Ube2W (pink). **b** Close-up of the E2:E3 interface. **c**, **d** Structural models of a RING:Ube2W~Ub complex with Ub in an open (**c**) or closed (**d**) conformation respectively. Based on superposition with the Ub-RING:Ube2N~Ub:Ube2V2 structure 7BBD^39^. In the closed conformation (**d**; boxed), Ub K11 makes a potential salt bridge with TRIM21 E13 and buries I44 at an interface with the E2. **e** Ube2W-mediated TRIM21 RING mono-ubiquitination assay using 2 µM R-NbGFP and 1 µM Ube2W with WT, I44A or K11E ubiquitin. Representative example from *n* = 2 independent experiments. **f** Schematic of antibody-induced recruitment of either two RINGs or two RING dimers. Only the latter satisfies the ‘two-plus-one’ model for RING autoubiquitination^20,39^. **g** Ube2W-mediated mono-ubiquitination assay using 10 µM R-PS or R-R-PS and 0.25 µM Ube2W WT or monomeric V30K/D67K. Representative example from *n* = 2 independent experiments. **h** Ubiquitination of 100 nM R-PS or R-R-PS in the absence or presence of equimolar anti-GFP antibody. Ube2W was titrated (25, 50, 100, 200 nM). Representative example from *n* = 3 independent experiments. **i** Quantification of monoubiquitination from (**h**). Graph shows mean and s.e.m. from *n* = 3 independent experiments. Statistical significance based on two-tailed Student’s *t* test (two-tailed). Source data are provided as a Source Data file.

To understand how TRIM21 recruits Ube2W, we solved the crystal structure of the RING domain in complex with a Ube2W dimerization mutant^46^ in which the active site cysteine was also replaced with lysine (Ube2W^V30K/D67K/C91K^). Two copies of each Ube2W and RING could be found in the asymmetric unit, with the two RINGs forming a homodimer as described previously^14,31,39^ (Fig. 1a). RING and Ube2W engage each other via the canonical RING:E2 interface (Fig. 1b). When RING E3s engage a ubiquitin-charged E2 they can adopt either an ‘open’ conformation, in which the ubiquitin is free to sample an ensemble of conformations^47^, or a ‘closed’ conformation in which the ubiquitin is in direct contact with both the E2 and E3^48^. We generated models using our RING:Ube2W structure where the ubiquitin is either dissociated (open; Fig. 1c) or in direct contact (closed; Fig. 1d) by superposing on the previously determined Ub-RING:Ube2N~Ub:Ube2V2 structure^39^. In our closed conformation model, we observed hydrophobic burial of ubiquitin residue I44, which is associated with a closed state^49^, and an interaction between ubiquitin residue K11 and RING E13. To determine if TRIM21 RING catalyses Ube2W ubiquitination using a closed conformation we mutated each of these interactions and determined their impact on in vitro ubiquitination activity using a previously described TRIM21 RING-nanobody construct (R-NbGFP)^15^. Mutation of I44A has previously been shown to inhibit ubiquitination by the E2 Ube2N^50^, which adopts a closed conformation^31^. In contrast, I44A has no observable effect on FANCL^UR^-Ube2T catalysis, indicating it does not require a closed conformation^51^. In our experiment, R-NbGFP efficiently catalyzed monoubiquitination using Ube2W and WT ubiquitin, but ubiquitin mutants I44A and K11E both abolished catalysis (Fig. 1e). This indicates that TRIM21 RING uses a closed conformation when catalyzing ubiquitination with Ube2W, as was found with Ube2N^39^. It has previously been shown that RING dimerization increases TRIM-mediated ubiquitination via both Ube2N^15,16,52,53^ and Ube2W^54^. Taken together, this suggests that TRIM21 RING utilizes a similar mechanism to catalyze ubiquitination by the different E2s, involving both a closed conformation and a RING dimer.

The importance of E13 in TRIM21 catalysis of Ube2W-mediated ubiquitination is of interest as this residue is part of a tri-ionic motif that was identified to drive interaction with Ube2N~Ub^31^. We tested whether other residues in this motif are involved in Ube2W binding by performing NMR titrations of ^15^N-labelled TRIM21 RING tri-ionic mutants against Ube2W^V30K/D67K/C91K^. Mutation of the tri-ionic residues E12 and E13 to alanine did not lead to obvious reductions in the observed chemical shift perturbations (CSPs) (Supplementary Fig. 1a–c). Moreover, tri-ionic mutants had only a modest effect on TRIM21 RING monoubiquitination (Supplementary Fig. 1d). TRIM21 RING mutants E13A and E13R both showed a slight reduction in activity, suggesting that residue E13 could indeed interact with ubiquitin K11, but this is not as critical as for Ube2N~Ub^31^. When comparing the RING in the RING:Ube2W structure to the apo^14^ and Ube2N~Ub^31^ -engaged structures, we noted that the N- and C-terminal helices of the RINGs are partly unfolded when bound by Ube2W (Supplementary Fig. 1e). While this may reflect differences in crystallization, it could suggest that Ube2W has the potential to destabilize the 4-helix bundle, thereby generating a disordered N-terminus for modification^55^. Even so, this is insufficient to explain how Ube2W can modify the N-terminus of a RING it is bound to, as it is located far away from the E2 active site.

### A Ube2W dimer monoubiquitinates TRIM21 RING

We postulated that as Ube2W is normally dimeric^46^, it may utilize a similar catalytic RING topology to that previously described for the Ube2N/Ube2V2 heterodimer^39^. Under such an arrangement, two RINGs could form a dimer to act as the enzyme, activating the donor ubiquitin on one Ube2W monomer, while a third RING acts as the substrate, oriented by the second Ube2W monomer to allow attack on the N-terminus. To test this hypothesis, we took advantage of two previously-characterized TRIM21 constructs carrying either one (R) or two (R-R) RINGs and a PRYSPRY (PS) domain (R-PS and R-R-PS) that allow precise control of the number of RING domains recruited in close proximity upon antibody binding (Fig. 1f)^39^. We found that both constructs were capable of catalyzing monoubiquitination by Ube2W, however this activity was abolished when monomeric Ube2W^V30K/D67K^ was used (Fig. 1g). This is consistent with Ube2W dimerization being used to orient one RING domain as a substrate. Surprisingly, no difference between R-PS and R-R-PS was observed, probably because the relatively high concentrations were sufficient to drive RING dimerization of R-PS. Previously we have shown that TRIM21 is activated in cells by substrate-induced clustering^15^ and indeed the addition of IgG Fc to in vitro ubiquitination experiments with the R-R-PS construct is required to induce K63-chain formation by Ube2N/Ube2V2 under near-physiological enzyme concentrations^39^. We therefore reduced R-PS or R-R-PS concentrations and titrated Ube2W either in the presence or absence of antibody. Under these conditions, monoubiquitination was only observed with R-R-PS. Moreover, R-R-PS monoubiquitination was greatly stimulated by the addition of antibody (Fig. 1h, i)^15,39^. Taken together, these data suggest that antibody binding promotes TRIM21 RING monoubiquitination by dimeric Ube2W through a similar *trans* mechanism to Ube2N/Ube2V2.

### Biochemical inhibition of TRIM21 RING N-terminal ubiquitination

**Fig. 2:**
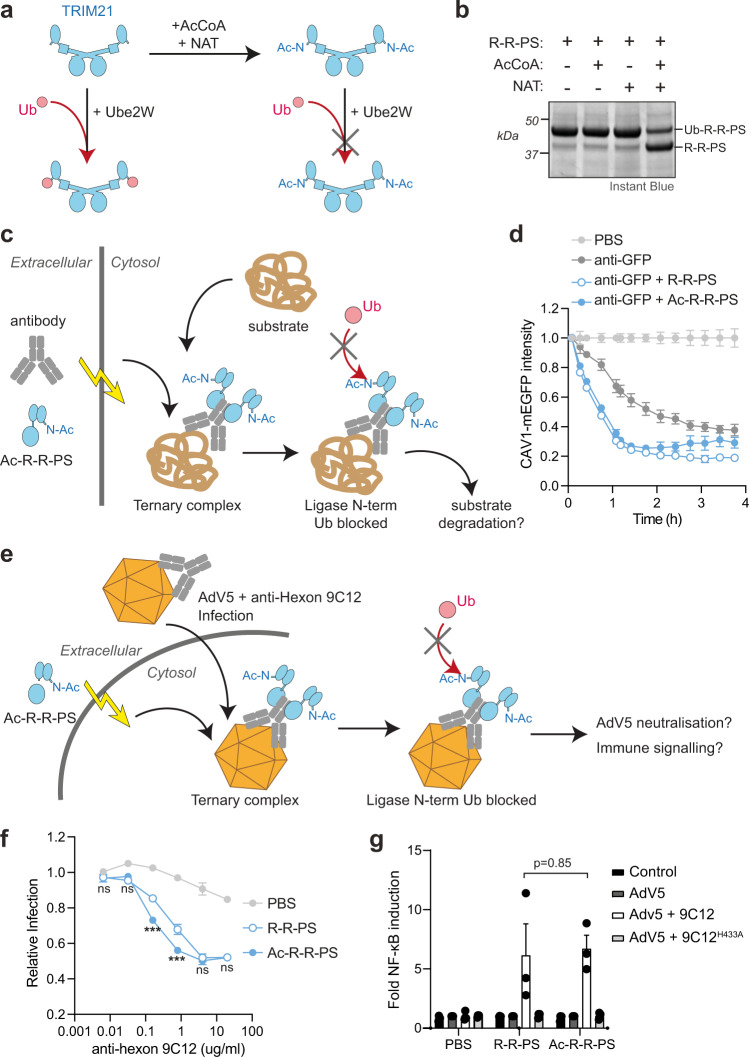
Ligase N-terminal ubiquitination is not required for substrate degradation and antiviral activity. **a** Schematic showing N-terminal acetylation of TRIM21 by AcCoA and NAT and then incubation of the acetylated or unmodified ligase with ubiquitin (Ub) and Ube2W in a ubiquitination reaction. **b** Protein-stained gel of ubiquitination reaction depicted in (**a**) using R-R-PS ligase. Monoubiquitination of R-R-PS that has been incubated with AcCoA and NAT is inhibited. Representative example from *n* = 3 independent experiments. **c** Schematic showing Trim-Away experiment in which antibody is electroporated into cells together with Ac-R-R-PS. Once inside cells, a ternary complex with the substrate is formed. If degradation is driven by ligase N-terminal ubiquitination, then Ac-R-R-PS activity will be inhibited. **d** Results of Trim-Away experiment described in (**c**). RPE-1 CAV1-mEGFP cells were electroporated with PBS or anti-GFP antibody ± R-R-PS proteins and CAV1-mEGFP fluorescence was quantified using the IncuCyte system. Time shows hours (h) post-electroporation. Values normalized to PBS control condition. Graphs shows mean and s.e.m. from *n* = 4 technical replicates. Representative example from *n* = 2 independent experiments. Note that there is CAV1-mEGFP degradation with anti-GFP alone due to the presence of endogenous cellular TRIM21. **e** Schematic showing electroporation of Ac-R-R-PS into cells followed by infection with Adv5 in the presence of anti-hexon antibody 9C12. If ligase N-terminal ubiquitination is necessary for TRIM21 antiviral function, neutralization of infection and immune signaling will be inhibited. **f**, **g** Neutralization of AdV5 infection by increasing 9C12 concentrations (**f**) and AdV5-9C12-induced NFκB activation (**g**) in HEK293T TRIM21 KO cells infected immediately after electroporation with PBS or R-R-PS ± acetylation. 9C12^H433A^ does not bind TRIM21 PRYSPRY. Graphs shows mean and s.e.m. from *n* = 3 independent experiments. Black dots in (**g**) show individual data points. Statistical significance between R-R-PS and Ac-R-R-PS is based on two-way ANOVA (**f**; significance is represented with labels ns (not significant, *P* > 0.05), *** (*P* ≤ 0.001)) and two-tailed Student’s *t* test (**g**). Source data are provided as a Source Data file.

To investigate the requirement for TRIM21 RING N-terminal monoubiquitination for TRIM21 cellular function, we decided to block it biochemically via N-terminal acetylation. N-terminal acetylation is an irreversible modification catalyzed in cells by N-Acetyl Transferases (NATs) using the co-factor Acetyl-CoA^56^. We chose Naa50, a NAT from *Chaetomium thermophilum*, as its substrate specificity (MASS, MVNA) matches the N-terminus of TRIM21 (MASA), and it displays thermostability and high in vitro activity^57,58^. The R-R-PS construct was used to allow functional readout of N-terminal acetylation by in vitro ubiquitination assays with Ube2W (Fig. 2a). Incubation of NAT with R-R-PS in the presence of Acetyl-CoA resulted in successful N-terminal acetylation as confirmed by LC-MS/MS (Supplementary Fig. 2a). N-terminally acetylated R-R-PS (Ac-R-R-PS) was added together with Ube2W and ubiquitin to test whether monoubiquitination was inhibited (Fig. 2a). In the absence of acetylation, all R-R-PS was monoubiquitinated, whereas R-R-PS incubated with both NAT and Acetyl-CoA remained predominantly non-ubiquitinated (Fig. 2b). The degree of monoubiquitination inhibition was proportional to the time of NAT and Acetyl-CoA incubation (Supplementary Fig. 2b). Importantly, the formation of free K63-linked ubiquitin chains was not compromised, demonstrating that the acetylated RING remains catalytically active (Supplementary Fig. 2c). These results confirm both that TRIM21 RING is monoubiquitinated by Ube2W at its N-terminus and that this can be inhibited by N-terminal acetylation.

### TRIM21 RING N-terminal ubiquitination is not required for its cellular function

With a method to specifically block N-terminal ubiquitination of TRIM21 RING, we tested whether this is required for TRIM21 cellular function. We used two model substrates to assay for TRIM21 function; Adenovirus 5 (Adv5) targeted with an anti-Hexon antibody (Adv5 neutralization) and GFP-tagged Caveolin-1 (CAV1-mEGFP) targeted with an anti-GFP antibody (Trim-Away). The multi-epitope (Adv5^59,60^) and oligomeric (CAV1^61^) nature of these substrates induce TRIM21 clustering and its activation^15^. For the Trim-Away assay, either R-R-PS or Ac-R-R-PS were electroporated together with anti-GFP antibody into cells expressing CAV1-mEGFP and the kinetics of degradation monitored over time (Fig. 2c). Consistent with previous Trim-Away experiments, electroporation of anti-GFP antibody drove CAV1-mEGFP degradation via endogenous TRIM21 (Fig. 2d, dark gray). However, CAV1-mEGFP degradation was substantially accelerated through the delivery of exogenous R-R-PS (Fig. 2d, closed blue circles). Importantly, ligase N-terminal acetylation (Ac-R-R-PS) had no impact and CAV1-mEGFP degradation proceeded with identical kinetics (Fig. 2d, open blue circles). Next, we tested whether TRIM21 RING N-terminal acetylation was required for intracellular antibody-mediated Adv5 neutralization. TRIM21 knockout cells were electroporated with either R-R-PS or Ac-R-R-PS, then infected with AdV5 in the presence of the anti-hexon antibody 9C12 (Fig. 2e). Previous experiments have shown that when TRIM21 binds to antibody-coated virus it blocks infection by mediating proteasomal-degradation of the virion^2^. Electroporation of R-R-PS could rescue TRIM21 knockout and neutralized AdV5 in an antibody-dose dependent manner (Fig. 2f). Ac-R-R-PS was at least as active as R-R-PS at neutralizing AdV5 infection and at intermediate antibody concentrations was more effective (Fig. 2f). In addition to mediating virion degradation, TRIM21 also activates innate immune signalling upon detection of antibody-coated virus^30^. Both R-R-PS and Ac-R-R-PS were equally effective at stimulating NF-κB-driven transcription in response to antibody-coated Adv5 (Fig. 2g). This was dependent upon direct engagement of R-R-PS with antibody as the 9C12 mutant H433A, which specifically ablates binding to the PRYSPRY^21^, failed to activate NF-κB (Fig. 2g). Taken together, the data show that N-terminal ubiquitination of the RING ligase is dispensable for TRIM21 cellular function.

### TRIM21 RING N-terminal ubiquitination regulates its own stability

**Fig. 3:**
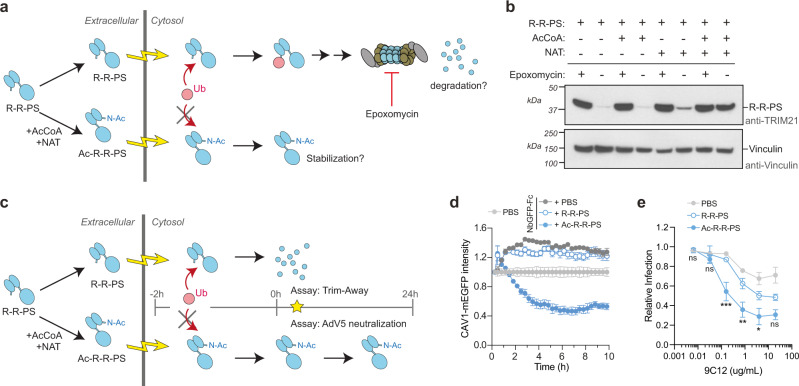
N-terminal ubiquitination regulates ligase turnover in cells. **a** Schematic showing electroporation of R-R-PS ± N-terminal acetylation and proteasome-dependent degradation. **b** Western blot of experiment depicted in (**a**) 1 h post-electroporation. R-R-PS protein levels are rescued by addition of 10 µM proteasome inhibitor epoxomycin. Ac-R-R-PS protein persists in cells irrespective of proteasome inhibition. Representative example from *n* = 2 independent experiments. **c** Schematic showing electroporation of R-R-PS ± acetylation into cells, followed by delayed Trim-Away or Adv5 neutralization assays. **d** For the delayed Trim-Away assay, mRNA encoding the antibody construct (NbGFP-Fc) responsible for recruiting R-R-PS to substrate (CAV1-mEGFP) was co-electroporated into NIH3T3 CAV1-mEGFP cells with PBS or R-R-PS ± acetylation; Trim-away is delayed for ~2 h until NbGFP-Fc protein is translated. Graph shows mean and s.e.m. from *n* = 4 technical replicates of CAV1-mEGFP fluorescence quantified using the IncuCyte system. Values normalized to PBS control condition (no NbGFP-Fc). Time shows hours (h) post-electroporation. Representative example from *n* = 2 independent experiments. Note that NIH3T3 cells do not contain endogenous TRIM21 and expression of NbGFP-Fc in the absence of TRIM21 activity leads to CAV1-mGFP stabilization. **e** For the delayed Adv5 neutralization assay, HEK293T TRIM21 KO cells were infected with AdV5 ± 9C12 2 h post-electroporation of PBS or R-R-PS ± acetylation. Graph shows mean and s.e.m. from *n* = 3 independent experiments. Statistical significance between R-R-PS and Ac- R-R-PS is based on two-way ANOVA and represented with labels ns (not significant, *P* > 0.05), * (*P* ≤ 0.05), ** (*P* ≤ 0.01), *** (*P* ≤ 0.001). See also Supplementary Fig. 3. Source data are provided as a Source Data file.

We reasoned that if RING ligase N-terminal ubiquitination is not required for substrate degradation, perhaps it is involved in mediating self-turnover. We therefore electroporated Ac-R-R-PS into cells and monitored protein levels after 1 h (Fig. 3a). Comparison of epoxomicin-treated and untreated cells revealed that non-acetylated R-R-PS was readily degraded by the proteasome (Fig. 3b, first two lanes). In contrast, epoxomicin had little impact on Ac-R-R-PS protein levels, indicating that blocking ligase N-terminal ubiquitination prevents its proteasomal degradation (Fig. 3b, last two lanes). R-R-PS degradation was mediated only partially though its own RING ligase activity (Supplementary Fig. 3a,b), thus additional regulatory mechanisms such as N-end rule-mediated degradation may also act on the R-R-PS N-terminus. The ligase-dead (I18R/M72E) R-R-PS, despite rapid turnover, could no longer degrade substrate in a Trim-Away assay (Supplementary Fig. 3c), suggesting that degradation of ligase and substrate can be uncoupled even when in a ternary complex with antibody.

While the data suggests that ligase N-terminal ubiquitination is not required for substrate degradation, it may be used to regulate ligase levels. To test this, we repeated our electroporation experiments with R-R-PS or Ac-R-R-PS but measured either Trim-Away or AdV5 neutralization activity after a delay of several hours (Fig. 3c). For Trim-Away experiments, this was accomplished by co-electroporating mRNA encoding the antibody construct responsible for recruiting R-R-PS to CAV1-mEGFP (NbGFP-Fc). Trim-Away is thus delayed by several hours while NbGFP-Fc is expressed. Under this experimental regime, there was no longer any substrate degradation in cells electroporated with R-R-PS (Fig. 3d, compare with Fig. 2d). In contrast, Ac-R-R-PS degraded CAV1-mEGFP just as efficiently as when Trim-Away proceeds immediately upon ligase electroporation (Fig. 3d, compare with Fig. 2d). For AdV5 neutralization experiments, cells were infected 2 h post-ligase delivery. In this case, neutralization by R-R-PS neutralization was severely attenuated with Ac-R-R-PS inhibiting infection significantly more efficiently (Fig. 3e).

### In vitro Trim-Away reveals direct substrate ubiquitination

**Fig. 4:**
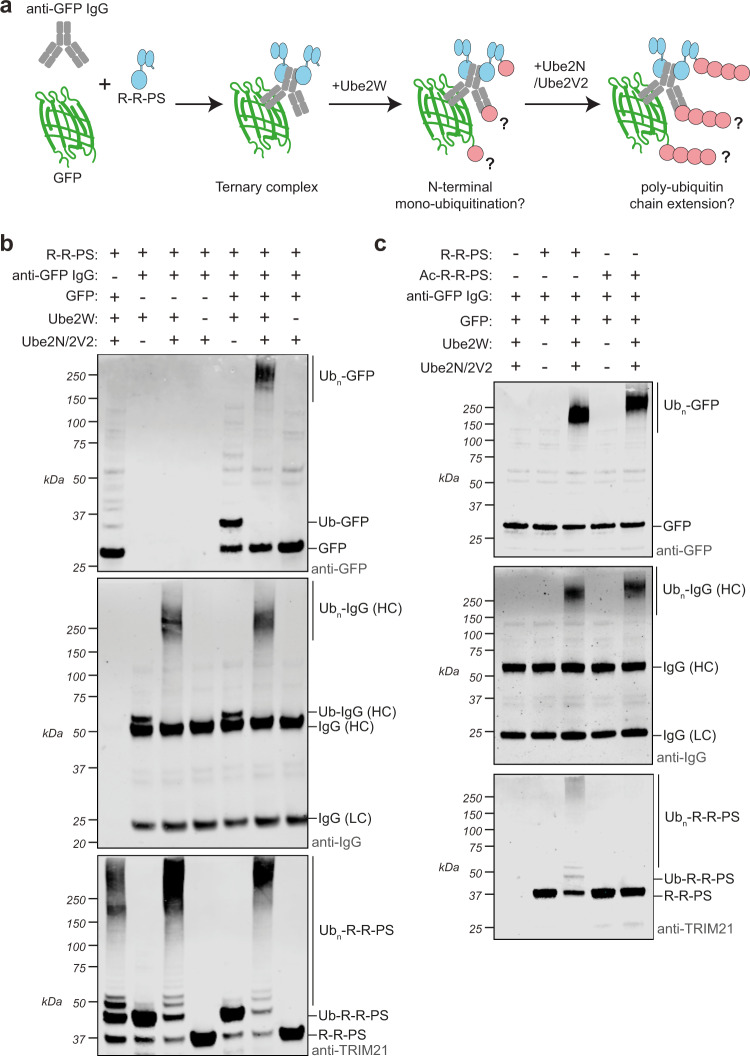
In vitro Trim-Away reveals direct substrate ubiquitination. **a** Schematic showing an in vitro ubiquitination reaction in which ligase (R-R-PS), substrate (GFP) and anti-GFP antibody are incubated together with E2 enzymes Ube2W and Ube2N/Ube2V2 to promote either mono- or polyubiquitination. **b** Western blot of experiment described in (**a**). Top panel is blotted for GFP, middle panel for IgG and lower panel for TRIM21. Depending on the E2s present, monoubiquitinated species or a higher molecular weight smear indicative of polyubiquitin are observed. Representative example from *n* = 2 independent experiments. **c** Western blot of experiment similar to (**b**) but comparing R-R-PS to Ac-R-R-PS. Note that while R-R-PS ubiquitinates itself, antibody heavy chain and substrate, Ac-R-R-PS only ubiquitinates antibody and substrate. Representative example from *n* = 2 independent experiments. Source data are provided as a Source Data file. See also Supplementary Fig. 4.

As ligase autoubiquitination is not required for substrate degradation, we investigated whether this might be driven by substrate ubiquitination instead. To test this, we performed in vitro ubiquitination experiments with R-R-PS, anti-GFP antibody and recombinant GFP substrate (Fig. 4a). We observed simultaneous ubiquitination of R-R-PS, antibody heavy chain and GFP; monoubiquitination was observed upon incubation with Ube2W alone, whereas in conditions where Ube2N/Ube2V2 was also included this resulted in anchored polyubiquitin chains (Fig. 4b). Importantly, GFP ubiquitination only occurred when both R-R-PS and antibody were also present (Fig. 4b and Supplementary Fig. 4). This is consistent with the requirement for antibody to recruit the R-R-PS ligase to its substrate. Next, we asked whether substrate ubiquitination occurs independently of ligase ubiquitination or if the latter modification is required to stimulate catalytic activity. To do this, we repeated our in vitro ubiquitination assay in the presence of both E2s and compared R-R-PS with Ac-R-R-PS. Ligase N-terminal acetylation blocked autoubiquitination but did not interfere with polyubiquitination of either antibody heavy chain or GFP (Fig. 4c). This data shows that TRIM21 RING catalyses polyubiquitination of an antibody-bound substrate and that this can occur independently of ligase autoubiquitination. Nevertheless, the fact that TRIM21, antibody and substrate can be simultaneously polyubiquitinated in vitro is consistent with in-cell Trim-Away data showing that all three components are simultaneously degraded^25^.

### Substrates are ubiquitinated during Trim-Away in live cells

**Fig. 5:**
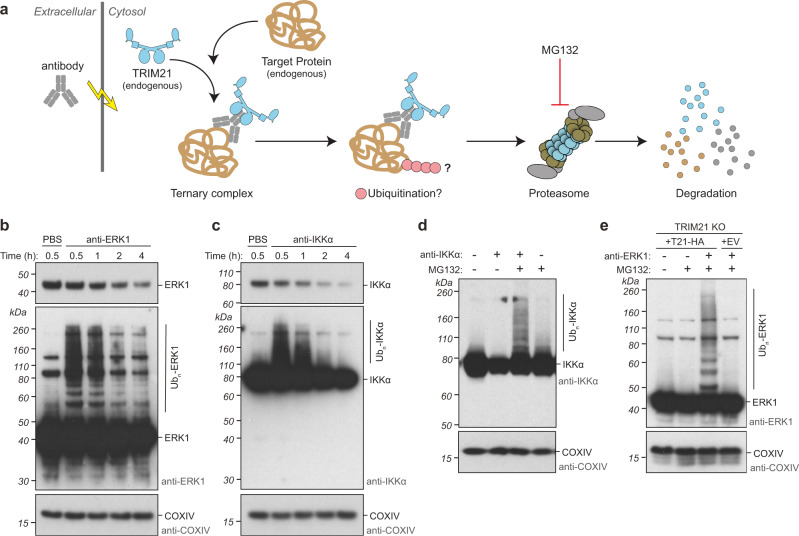
Substrate ubiquitination parallels substrate degradation during Trim-Away in cells. **a** Schematic showing Trim-Away experiment in which antibodies are electroporated into cells expressing endogenous TRIM21. Ubiquitination and degradation are then monitored in the presence or absence of proteasome inhibitor MG132. **b**, **c** RPE-1 cells were electroporated with PBS or anti-ERK1 antibody (**b**) or anti-IKKα antibody (**c**) and whole cell lysates harvested at the indicated times after electroporation for immunoblotting. Short exposures show degradation of substrates. Long exposures reveal substrate ubiquitination followed by degradation of ubiquitinated species. **d** RPE-1 cells were electroporated with PBS or anti-IKKα antibody ± MG132 and whole cell lysates harvested 1 h post-electroporation for immunoblotting. **e** RPE-1 TRIM21 KO cells reconstituted with TRIM21-HA (T21-HA) or empty vector (EV) were electroporated with PBS or anti-ERK1 antibody ± MG132 and whole cell lysates harvested 1 h post-electroporation for immunoblotting. Representative examples (**b**–**e**) from *n* = 3 independent experiments. Source data are provided as a Source Data file. See also Supplementary Fig. 5.

Next, we attempted to monitor substrate ubiquitination during Trim-Away mediated by endogenous TRIM21 in living cells (Fig. 5a). We chose two endogenous protein substrates, ERK1 and IKKα, and blotted for protein levels at various timepoints post-antibody electroporation. For both substrates, high-molecular weight bands or smearing consistent with polyubiquitination was observed at 30 min post-antibody electroporation (Fig. 5b, c). These high-molecular weight bands decreased over the next few hours simultaneous with a reduction in substrate protein levels (Fig. 5b, c). To obtain further evidence for substrate polyubiquitination we repeated the IKKα Trim-Away experiment in the presence of proteasome inhibitor MG132. Addition of MG132 had no impact in control cells, but in the presence of electroporated antibody higher molecular weight laddering was clearly observed (Fig. 5d). When performed as a time-course experiment, this revealed that IKKα ubiquitinated species first formed and then was depleted coincident with protein degradation. Treatment with MG132 both blocked degradation and led to a steady accumulation of ubiquitinated material (Supplementary Fig. 5e). To test whether antibody-dependent substrate ubiquitination is mediated by TRIM21 we repeated our experiments in TRIM21 knockout (TRIM21 KO) cells reconstituted with either HA-tagged TRIM21 (+T21-HA) or an empty vector (+EV) control. Antibody-induced ERK1 laddering indicative of polyubiquitination was observed in knockout cells reconstituted with TRIM21-HA but not empty vector (Fig. 5e). We also probed for TRIM21 and antibody in the above experiments, but did not observe obvious ubiquitinated species, despite concomitant TRIM21 degradation (Supplementary Fig. 5a–d), perhaps because a smaller fraction of total TRIM21 and antibody are ubiquitinated compared to substrate, or TRIM21 ubiquitinated species are processed by DUBs^29^. Altogether these data indicate that substrates are ubiquitinated during Trim-Away in live cells in an antibody- and TRIM21-dependent manner.

Previously, we have shown that Trim-Away can be performed in the absence of antibody by fusing a substrate-targeting nanobody directly to the RING domain of TRIM21^15^. To confirm this approach accurately recapitulates endogenous TRIM21 function, we tested if a TRIM21 RING-nanobody fusion protein (R-NbGFP) also uses a substrate-induced clustering mechanism^15^. Indeed, similar to endogenous TRIM21, R-NbGFP could degrade oligomeric (CAV1-mEGFP-Halo) but not monomeric (mEGFP-Halo) substrate (Supplementary Fig. 6a,b). We therefore used this RING-nanobody approach to determine whether RING ligase activity of TRIM21 is required for substrate ubiquitination. Introducing two RING-inactivating mutations (I18R and M72E) into the R-NbGFP construct (R^I18R/M72E^-NbGFP)^15^ completely abolished both substrate ubiquitination and degradation (Supplementary Fig. 6c) suggesting these processes are driven by TRIM21 RING catalytic activity.

### Neither N-terminal nor lysine ubiquitination of TRIM21 RING is required for Trim-Away

**Fig. 6:**
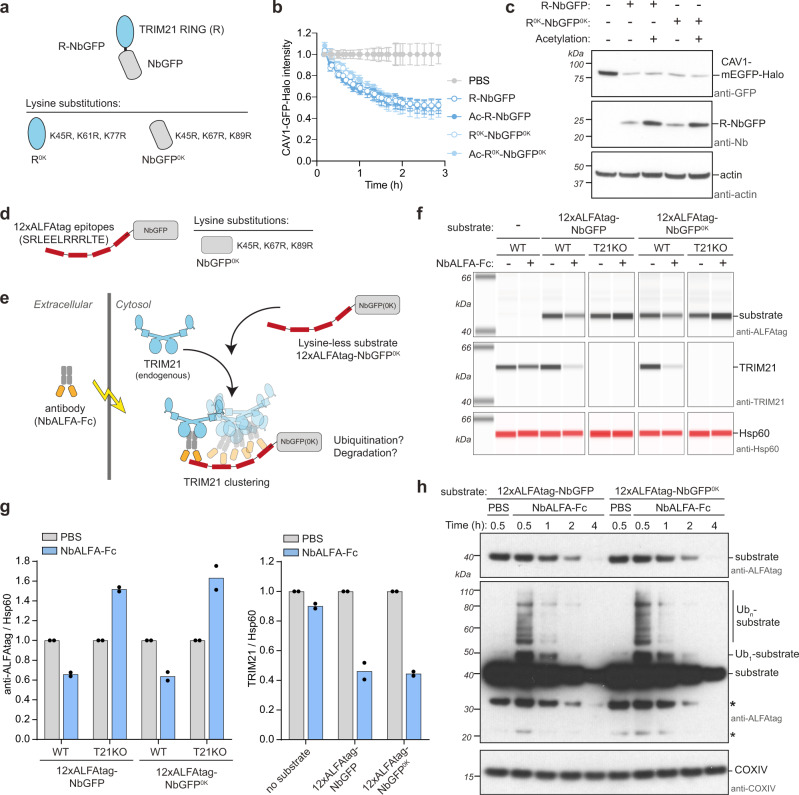
Trim-Away ubiquitinates and degrades a lysine-less substrate. **a** Schematic showing R-NbGFP construct and substitutions to remove all lysines. **b**, **c** RPE-1 CAV1-mEGFP-Halo cells were electroporated with PBS or R-NbGFP ± lysines ± N-terminal acetylation. **b** CAV1-mEGFP-Halo fluorescence was quantified using the IncuCyte system. Time shows hours (h) post-electroporation. Values normalized to PBS control condition. Graphs shows mean and s.e.m. from *n* = 4 technical replicates. **c** Whole cell lysates were harvested 3 h post-electroporation for immunoblotting. Representative examples (**b**, **c**) from *n* = 3 independent experiments. **d** Schematic showing a model lysine-less substrate consisting of NbGFP (with lysine substitutions) fused to 12 copies of the naturally lysine-less ALFAtag epitope. **e** Scheme showing Trim-Away experiment in which the anti-ALFAtag antibody (NbALFA-Fc) is electroporated into cells expressing endogenous TRIM21 and the lysine-less substrate (12xALFAtag-NbGFP^0K^). **f**, **g** RPE-1 WT or TRIM21 KO cells expressing either substrate with lysines (12xALFAtag-NbGFP) or without lysines (12xALFAtag-NbGFP^0K^) were electroporated with PBS or NbALFA-Fc and whole cell lysates harvested 8 h post-electroporation for capillary-based immunoblotting. Lane view (**f**) and quantification (**g**) of substrate and TRIM21 protein levels normalized to PBS condition. Graphs show mean from *n* = 2 independent experiments (black dots). Note that binding of NbALFA-Fc in the absence of TRIM21 causes stabilization of substrate. **h** RPE-1 cells expressing either substrate with lysines (12xALFAtag-NbGFP) or without lysines (12xALFAtag-NbGFP^0K^) were electroporated with PBS or NbALFA-Fc and whole cell lysates harvested at the indicated times after electroporation for immunoblotting. Short exposures show degradation of substrates. Long exposures reveal substrate ubiquitination followed by degradation of ubiquitinated species. Representative example from *n* = 2 independent experiments. Single stars (*) show 8x- and 4x-ALFAtag-NbGFP species. Source data are provided as a Source Data file.

The preceding data show that activated TRIM21 RING can catalyze simultaneous ubiquitination of itself, antibody and substrate. However, blocking N-terminal TRIM21 RING ubiquitination does not prevent substrate degradation. To test if ligase lysine autoubiquitination is required for substrate degradation, we made a variant of R-NbGFP in which all lysines were substituted with arginines (Fig. 6a; R^K0^-NbGFP^K0^). Removal of all lysines from the ligase had no impact on either the kinetics or efficiency of substrate degradation (Fig. 6b, c). Furthermore, simultaneous blocking the N-terminus via acetylation also had no effect (Fig. 6b, c). Taken together, these data show that substrate degradation during Trim-Away is not dependent upon ligase N-terminal or lysine ubiquitination. Consistent with the in vitro ubiquitination assay (Fig. 4), inhibiting N-terminal autoubiquitination reduced ligase depletion without altering substrate degradation (Fig. 6c), suggesting that substrate and ligase turnover can be uncoupled.

### Trim-Away ubiquitinates and degrades lysine-less and N-terminally acetylated substrates

To test whether Trim-Away is driven by substrate lysine ubiquitination, we designed model substrates for TRIM21 comprising a dodecameric ALFAtag repeat, which naturally contains no lysine residues^62^, fused to NbGFP with or without lysines (Fig. 6d). These substrates were expressed in either wild-type (WT) or TRIM21 knockout (T21KO) cells, which were then electroporated with an anti-ALFAtag antibody (NbALFA-Fc). The NbALFA-Fc should bind the ALFAtag epitopes and recruit endogenous TRIM21 via the canonical PRYSPRY-Fc interaction, leading to TRIM21 clustering, activation and degradation (Fig. 6e). Indeed, NbALFA-Fc electroporation triggered substrate degradation in WT but not T21KO cells (Fig. 6f, g). Importantly, degradation was not dependent upon substrate lysines, as substrates both with and without lysines were equally well degraded (Fig. 6f, g). As expected for a Trim-Away experiment, endogenous TRIM21 was also degraded alongside each substrate (Fig. 6f, g). Next, we addressed whether substrates with and without lysines show a similar pattern of ubiquitination during their degradation. To this end we expressed the substrates in WT cells (endogenous TRIM21) and blotted for protein levels at timepoints post-electroporation of NbALFA-Fc. Both substrates were degraded with similar kinetics within 4 h of antibody delivery. Strikingly, identical high-molecular weight bands and smearing indicative of polyubiquitination were observed at 30 min post-electroporation regardless of the presence of lysines in the substrate (Fig. 6h). These bands disappeared over time concomitant with reduced substrate protein levels (Fig. 6h). Thus, TRIM21 causes efficient substrate ubiquitination and degradation and does not require substrate lysines.

**Fig. 7:**
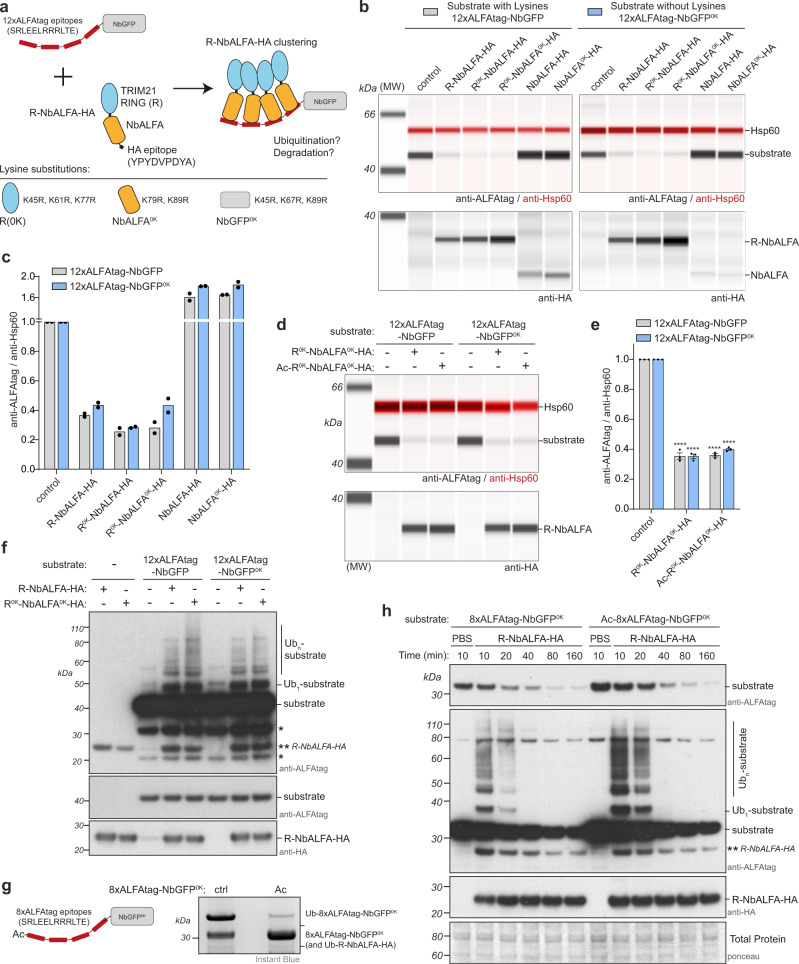
Trim-Away independently of lysine and N-terminal ubiquitination. **a** Schematic of completely lysine-less Trim-Away assay. The substrate constitutes NbGFP with 12 copies of the ALFAtag epitope. The ligase constitutes the TRIM21 RING fused to NbALFA (R-NbALFA). The substitutions necessary to remove lysines from each domain in the assay are shown. Note that the ALFAtag and HA epitopes are naturally lysine-less. The 12xALFAtag allows clustering of multiple R-NbALFA molecules, triggering RING activation. **b–e** RPE-1 TRIM21 KO cells expressing either substrate with lysines (12xALFAtag-NbGFP) or without lysines (12xALFAtag-NbGFP^0K^) were electroporated with water (control) or mRNA encoding the indicated constructs (**b**, **c**) or PBS (control) or R^0K^-NbALFA^0K^-HA protein ± acetylation (**d**, **e**) and whole cell lysates harvested 8 h (**b**, **c**) or 3 h (**d**,**e**) post-electroporation for capillary-based immunoblotting. Lane view (**b**, **d**) and quantification (**c**, **e**) of substrate protein levels normalized to control condition. Graphs shows mean (**c**) or mean and s.e.m. (**e**) from *n* = 2 (**c**) or *n* = 3 (**e**) independent experiments (black dots). Statistical significance from control condition is based on two-way ANOVA and represented with label **** (*P* ≤ 0.0001). Note that binding of NbALFA alone causes stabilization of substrate. **f** RPE-1 TRIM21 KO cells expressing either substrate with lysines (12xALFAtag-NbGFP) or without lysines (12xALFAtag-NbGFP^0K^) were electroporated with ligase R-NbALFA ± lysines in the presence of MG132 and whole cell lysates harvested 30 min post-electroporation for immunoblotting. Single stars (*) show 8x- and 4x-ALFAtag-NbGFP species. Representative example from *n* = 2 independent experiments. **g** Lysine-less substrate 8xALFAtag-NbALFA^0K^ with (Ac) or without (ctrl) N-terminal acetylation was incubated with ligase R-NbALFA and Ube2W in a ubiquitination reaction. Representative example from *n* = 2 independent experiments. **h** RPE-1 TRIM21 KO cells were electroporated with substrate 8xALFAtag-NbGFP^0K^ ± acetylation and 4 h later electroporated with PBS or ligase R-NbALFA protein and whole cell lysates harvested at the indicated times after electroporation for immunoblotting. Short exposures show degradation of substrates. Long exposures reveal substrate ubiquitination followed by degradation of ubiquitinated species. Representative example from *n* = 2 independent experiments. Source data are provided as a Source Data file.

As it is formally possible that ligase lysine ubiquitination may influence substrate ubiquitination, we modified our assay to remove lysines simultaneously from both ligase and substrate. To do this, we complemented our model substrate with a model ligase comprising a TRIM21 RING fused to the anti-ALFAtag nanobody^62^ (R-NbALFA; Fig. 7a). TRIM21 RING-nanobody fusion proteins employ a substrate-induced clustering mechanism identical to endogenous TRIM21 (Supplementary Fig. 6). Thus, in this assay the ALFAtag substrate should recruit multiple R-NbALFA ligases, leading to ligase clustering, activation and degradation (Fig. 7a). Indeed, as with endogenous TRIM21, recruitment of R-NbALFA to substrates both with and without lysines led to their efficient degradation (Fig. 7b, c). However, Trim-Away was equally efficient when both ligase and substrate were lysine-less (Fig. 7b, c). Lysine-less Trim-Away was also unaffected by blocking ligase N-terminal ubiquitination (Fig. 7d, e). Furthermore, the pattern of substrate ubiquitination induced by R-NbALFA was identical regardless of the presence of lysines (Fig. 7f). Thus, lysines are completely dispensable for Trim-Away to ubiquitinate and degrade substrates.

We next asked if ubiquitination of substrate N-terminus is responsible for its degradation. We generated a recombinant protein version of the lysine-less substrate (8xALFAtag-NbGFP^0K^) with an N-terminal MA- sequence to allow acetylation by NAT in vitro. N-terminal acetylation of 8xALFAtag-NbGFP prevented its ubiquitination by R-NbALFA utilizing Ube2W in vitro (Fig. 7g). We then delivered 8xALFAtag-NbGFP^0K^ ± N-terminal acetylation to TRIM21 KO cells and monitored protein levels at timepoints post-electroporation of R-NbALFA protein. The N-terminally acetylated substrate was degraded with similar kinetics to the non-acetylated control (Fig. 7h). Furthermore, both substrates showed a similar pattern of ubiquitination following R-NbALFA electroporation (Fig. 7h). Taken together, our data show that ligase autoubiquitination does not drive Trim-Away, but also that neither substrate lysine nor N-terminal ubiquitination are required.

## Discussion

Existing models of TRIM ligase ubiquitination for antivirals TRIM5 and TRIM21 propose that Ube2W-dependent N-terminal RING autoubiquitination drives substrate degradation^27,63^ (Supplementary Fig. 7). These models are based on data showing that substrate and ligase are co-degraded^23^, with identical kinetics^15,25^, and that while ligase autoubiquitination is readily detected in cells^15,26,29^, ubiquitination of viral substrates is not. Here we show that the TRIM21 RING domain interacts with ubiquitin-charged Ube2W and that mutating these interactions, or Ube2W dimerization, inhibits ligase autoubiquitination. We also show that Ube2W-dependent ligase autoubiquitination is stimulated by antibody binding, at least when using a R-R-PS construct where the requirements for RING clustering are satisfied without the need for an oligomeric substrate^15,39^. Ube2W is thought to modify N-termini rather than lysine side-chains because of its unique active site chemistry^55^. However, determining if TRIM21 N-terminal ubiquitination drives its degradation activity is challenging because, unlike with lysine ubiquitination, a genetic mutagenesis approach cannot be used. We therefore developed a biochemical method in which we produced recombinant TRIM21 RING-containing proteins, acetylated their N-termini in vitro to prevent their ubiquitination, and electroporated them into cells. We found that N-terminally acetylated TRIM21 RING-containing proteins were just as active as their non-acetylated counterparts in all cellular assays for TRIM21 function.

This N-terminal acetylation data contrasts with previous siRNA depletion experiments showing Ube2W knockdown reduces TRIM5 and TRIM21 antiviral functions^20,27,29^. The different results may be because the requirement for Ube2W-mediated TRIM21 RING autoubiquitnation is adenovirus-specific, although the ability of an N-terminally acetylated R-R-PS to neutralize adenovirus infection even more efficiently than a non-acetylated equivalent argues against this. It is important to note that the R-R-PS construct does not require the same substrate-induced clustering as full length TRIM21, so it could be that it makes use of a different ubiquitination and degradation pathway, although our in vitro experiments show that it remains stimulated by binding to antibody. The data presented here suggest that instead of relying on ligase autoubiquitination, Trim-Away mediates protein degradation by directly ubiquitinating substrates. However, a requirement for Ube2W to ubiquitinate substrate rather than ligase N-termini is also unlikely as N-terminally acetylated substrate was efficiently ubiquitinated and degraded. This finding is significant as it helps explain why Trim-Away can degrade diverse substrates even though the majority of cellular proteins are N-terminally acetylated^64^. While we cannot discount a role for Ube2W only when recruited by endogenous TRIM21, our results suggest that either Ube2W is not essential for Trim-Away or it is required for an activity that does not involve N-terminal ubiquitination^65^.

To determine what ubiquitination is required to drive TRIM21 ligase-dependent degradation, we created ligase and substrate constructs that lack lysine residues. Endogenous TRIM21 efficiently ubiquitinated a lysine-less substrate when recruited by antibody, leading to rapid substrate degradation. Indeed, there was no significant difference in either ubiquitination or degradation efficiency of substates with or without lysines, suggesting that lysines are not even a preferred target for TRIM21. Using the same lysine-less substrate in combination with lysine-less TRIM21 RING-nanobody ligase, we observed that substrate degradation was also unaffected by N-terminal acetylation of either the ligase or substrate. This suggests that N-terminal and lysine ubiquitination are not essential for substrate degradation by the TRIM21 RING E3.

These findings have important implications for TRIM ligase mechanism and pose two key questions; what modification drives substrate degradation and why does the ligase ubiquitinate and degrade itself? The answer to the latter question may lie in the differences between TRIM and CRL ligases. CRLs are assembled combinatorially from a small number of RINGs, adaptors and scaffolds^11,12^. In the absence of substrate, CRL complexes rapidly disassemble, providing an intrinsic functional shut-off^66^. In contrast, TRIM ligases encode catalytic and substrate binding domains in one polyprotein and so cannot be regulated by recruiting independent ligase components. Instead, TRIM proteins appear to be activated by forming higher-order structures^1,15,18^. The ability to form large oligomeric structures, sometimes called ‘cytoplasmic bodies’, is a common feature of TRIM proteins^67^. Cytoplasmic bodies can be induced simply by over-expression, likely because TRIMs have evolved to readily assemble into large scaffolds. These scaffolds activate TRIM RINGs by mediating dimerization and amplifying the ubiquitination signal associated with the ligase:substrate complex^15,20^. Evolving a ubiquitination mechanism whereby the ligase is ubiquitinated and co-degraded alongside its substrate may be a necessary step to ensure that, once-formed, large TRIM assemblies do not persist as cytoplasmic bodies inside the cell, continuously catalyzing ubiquitination. This may be particularly important for TRIMs like TRIM5 and TRIM21, which use their ubiquitin chains not only for degradation but also to potently stimulate inflammatory signalling^28,30^.

A regulatory role for ligase ubiquitination and degradation is further suggested by uncoupling it from substrate ubiquitination and degradation as we have demonstrated here. Previously, we have shown how a ‘two-plus-one’ catalytic RING topology drives TRIM autoubiquitination^20,29,39^. What drives substrate ubiquitination is currently unclear but may involve the quaternary structure unique to TRIM proteins. Despite their functional heterogeneity and inclusion of additional diverse domains, most TRIMs preserve not only the presence of a RING, B Box and coiled-coil but their relative arrangement and interdomain spacing^22^. The coiled-coils are antiparallel helices^13^ whose length is remarkably consistent across different TRIMs. This places each RING in a TRIM dimer at opposite ends of the molecule and a conserved distance apart. Many TRIMs also utilize a substrate-binding PRYSPRY domain and these are located centrally above the coiled-coil^68^. Given the fixed positions of these catalytic and substrate binding domains with respect to each other, it is tempting to speculate that this provides a similar catalytic topology as achieved by CRL ligases from separate components, with the coiled-coil playing an analogous role to a cullin protein.

Exactly how TRIM21 transfers ubiquitin to its substrate inside cells remains unclear. The fact that efficient substrate ubiquitination and degradation occurs when both N-terminal and lysine ubiquitination are prevented, suggests that degradation may proceed via non-canonical ubiquitination of non-lysine residues such as serines and threonines as has been reported for several E3 ligases^69^. Unlike most E3 ligases, TRIM21 does not have a cognate substrate or set of substrates. Instead, TRIM21 has evolved to hijack the diversity of the body’s antibody repertoire to degrade any antibody-bound protein, within which the availability of potential ubiquitination sites may significantly differ. This has some striking similarities to endoplasmic reticulum (ER)-associated degradation (ERAD), where only a few E3 ligase can ubiquitinate any of the thousands of different proteins that may fail to fold properly in the ER. In the case of ERAD E3 ligases, redundancy between lysine, serine and threonine ubiquitination is thought to allow their substrate promiscuity^70–73^. Whether a similar substrate ubiquitination site redundancy is employed by TRIM21 to degrade diverse substrates, and which E2 enzymes may facilitate this, remains to be explored.

## Methods

### Plasmids

A full list of plasmids used in this study, including primary sequences of all constructs can be found in Supplementary Data 1a. For lentivirus production, constructs were inserted into a modified version of pSMPP (Addgene #104970) where the SFFV promotor and puromycin resistance sequences were replaced with PGK1 promoter and Zeocin resistance sequences respectively (pPMEZ). For in vitro mRNA transcription, constructs were inserted into pGEMHE^74^, which contains UTR and polyA sequences for optimal mRNA stability and translation. For protein purification, constructs were inserted into derivations of the pOP and pET (Novagen) series of vectors.

### Lentivirus production

Lentivirus particles were collected from HEK293T cell supernatant 3 days after co-transfection (FuGENE 6, Promega) of lentiviral plasmid constructs (Supplementary Data 1a) with HIV-1 GagPol expresser pcRV1 (a gift from Dr. Stuart Neil) and pMD2G, a gift from Didier Trono (Addgene plasmid #12259). Supernatant was filtered at 0.45 µm before storage at −80 °C.

### In vitro transcription of mRNA

pGEMHE plasmid constructs (Supplementary Data 1a) were linearized and 5′-capped mRNA was synthesized with T7 polymerase (NEB HiScribeT7 ARCA kit) according to manufacturer’s instructions. mRNA concentration was quantified using a Qubit 4 fluorometer (ThermoFisher) and RNA Broad Range assay kit (ThermoFisher; Q10211).

### Protein expression and purification

A full list of purified proteins used in this study can be found in Supplementary Data 1b. Ube2W, Ube2N and Ube2V2, and TRIM21 RING, R-PS, R-R-PS, R-NbGFP, R^0K^-NbGFP^0K^ and mEGFP were expressed in *Escherichia coli* BL21 DE3. R-NbALFA, R^0K^-NbALFA^0K^, R^I18R/M72E^-R^I18R/M72E^-PS, and 8xALFAtag-NbGFP^0K^ were expressed in *Escherichia coli* C41. Ubiquitin and Ube1 were expressed in *E. coli* Rosetta 2 DE3 cells as previously described^39^. Cells were grown at 37 °C and 220 rpm until an OD^600^ of ~0.7. After induction, the temperature was reduced to 18 °C overnight. For TRIM21 and E2s induction was performed with 0.5 mM IPTG and 10 µM ZnCl_2_, for ubiquitin and Ube1 with 0.2 mM IPTG. mEGFP was expressed in ZY autoinduction media^75^ at 37 °C and 220 rpm. At OD^600^ of 0.7, the temperature was reduced to 18 °C for expression overnight. After centrifugation, cells were resuspended in 50 mM Tris pH 8.0, 150 mM NaCl, 10 µM ZnCl_2_, 1 mM DTT, 20% Bugbuster (Novagen) and Complete protease inhibitors (Roche, Switzerland). For His-tagged proteins, 20 mM imidazole was added to the buffer. Lysis was performed by sonication. R-PS and R-R-PS were expressed with N-terminal GST-SUMO tag and TRIM21^R^, Ube2W, Ube2V2 and Ube1 were expressed with N-terminal GST-tag followed by a TEV protease cleavage site and purified via glutathione sepharose resin (GE Healthcare) equilibrated in 50 mM Tris pH 8.0, 150 mM NaCl and 1 mM DTT. The tag was cleaved on beads overnight at 4 °C (with SUMO or TEV protease, respectively). Cleavage with SUMO protease resulted in no cleavage scar on R-PS and R-R-PS. TEV cleavage results in an N-terminal GSH-scar on TRIM21 RING, an N-terminal G-scar on Ube2N, an N-terminal GSQEF-scar on Ube2V2 and an N-terminal GSH-scar on Ube2W. In the case of Ube1, no protease cleavage was performed and the GST-Ube1 fusion protein was eluted using 50 mM Tris pH 8.0, 150 mM NaCl, 10 mM reduced glutathione and 1 mM DTT. mEGFP was expressed with an N-terminal His-tag without protease cleavage site and Ube2N was expressed with an N-terminal His-tag followed by a TEV protease cleavage site. R-NbGFP/NbALFA and 8xALFAtag-NbGFP^0K^ constructs were expressed as a His-SUMO fusion protein, to generate the native TRIM21 N-terminus after SUMO protease cleavage during purification. His-tagged proteins were purified via Ni-NTA resin equilibrated in 50 mM Tris pH 8.0, 150 mM NaCl, 20 mM imidazole and 1 mM DTT. Proteins were eluted in 50 mM Tris pH 8.0, 150 mM NaCl, 1 mM DTT, and 300 mM imidazole. For Ube2N, TEV-cleavage of the His-tag was performed overnight by dialyzing the sample against 50 mM Tris pH 8.0, 150 mM NaCl, 1 mM DTT, and 20 mM imidazole. Afterward, His-tagged TEV protease was removed by Ni-NTA resin. For R-NbGFP/NbALFA and 8xALFAtag-NbGFP^0K^ constructs SUMO protease cleavage was performed on the Ni-NTA resin overnight at 4 °C. Elution was performed on the next day using the equilibration buffer. Finally, size-exclusion chromatography of all proteins was carried out on either HiLoad 26/60 or 16/600 Superdex 75 prep grade column (GE Healthcare) in 20 mM Tris pH 8.0, 150 mM NaCl, and 1 mM DTT, except for GST-Ube1 for which either HiLoad 26/60 or 16/600 Superdex 200 prep grade column (GE Healthcare) were used. Ubiquitin purification was performed following the protocol established by the Pickart lab^76^. After cell lysis by sonication (lysis buffer: 50 mM Tris pH 7.4, 1 mg mL^−1^ Lysozyme (by Sigma Aldrich, St. Louis, USA), 0.1 mg mL^−1^ DNAse (by Sigma Aldrich, St. Louis, USA), a total concentration of 0.5% perchloric acid was added to the stirring lysate at 4 °C. The (milky) lysate was incubated for another 30 min on a stirrer at 4 °C to complete precipitation. Next, the lysate was centrifuged (19,500 *rpm*) for 30 min at 4 °C. The supernatant was dialyzed overnight (3500 MWCO) against 3 L 50 mM sodium acetate pH 4.5. Afterwards, Ub was purified via cation-exchange chromatography using a 20 mL SP column (GE Healthcare) using a NaCl gradient (0–1000 mM NaCl in 50 mM NaOAc pH 4.5). Finally, size-exclusion chromatography was carried out on a HiLoad 26/60 Superdex 75 prep grade column (GE Healthcare) in 20 mM Tris pH 7.4. Isotopically labelled proteins were expressed using *Escherichia coli* BL21 DE3 cells in M9 minimal media supplemented with ^15^NH_4_Cl (Sigma-Aldrich ISOTEC). *Chaetomium thermophilum* Naa50^82–289^ containing a C-terminal His-tag was expressed using *E. coli* Rosetta 2 cells in ZY autoinduction media^75^ which was grown at 37 °C and 220 rpm. At OD^600^ of 0.7, the temperature was reduced to 18 °C for expression overnight. *Ct*Naa50^82-289^ was purified as follows: Cells were harvested, resuspended in buffer A500 (20 mM HEPES pH 7.5, 500 mM NaCl, 20 mM imidazole) supplemented with a protease inhibitor mix (SERVA Electrophoresis GmbH, Germany) and lysed with a microfluidizer (M1-10L, Microfluidics). The lysate was cleared for 30 min at 50,000 g, 4 °C and filtered through a 0.45 µm membrane. The supernatant was applied to a 1 mL HisTrap FF column (GE Healthcare) for Ni-IMAC (immobilized metal affinity chromatography) purification. The column was washed with buffer A500 and the proteins were eluted with buffer A500 supplemented with 250 mM imidazole. *Ct*Naa50^82-289^ was subsequently purified by SEC (size-exclusion chromatography) using a Superdex 75 26/60 gel filtration column (GE Healthcare) in buffer G500 (20 mM HEPES pH 7.5, 500 mM NaCl). SUMO protease (MBP-Ulp1 (based on R3 sequence^77^) was purified using an MBPTrap HP 5 ml column and eluted with 50 mM Tris pH 8, 150 mM NaCl, 1 mM DTT and 10 mM Maltose. Finally, the eluted fractions were separated on a HiLoad 26/60 Superdex 75 pg SEC column (150 mM NaCl, 50 mM Tris pH 8 and 1 mM DTT).

### Cell culture

HEK293T (ATCC; CRL-3216) and NIH3T3-CAV1-EGFP^78^ cells were cultured in DMEM medium (Gibco; 31966021) supplemented with 10% calf serum and penicillin-streptomycin. RPE-1 cells (ATCC; CRL-4000) were cultured in DMEM/F-12 medium (Gibco; 10565018) supplemented with 10% Calf Serum and penicillin-streptomycin. All cells were grown at 37 °C in a 5% CO_2_ humidified atmosphere and regularly checked to be mycoplasma-free. The sex of NIH3T3 cells is male. The sex of HEK293T and RPE-1 cells is female. For proteasome inhibition experiments, MG132 (Sigma; C2211) was used at a final concentration of 25 µM and Epoxomicin (Sigma; 324801) was used at 10 µM. Following electroporation, cells were grown in medium supplemented with 10% calf serum without antibiotics. Live imaging was performed using the IncuCyte S3 live cell analysis system (Sartorius) housed within a 37 °C, 5% CO_2_ humidified incubator. For live imaging with the IncuCyte, cell culture medium was replaced with Fluorobrite (Gibco; A1896701) supplemented with 10% calf serum and GlutaMAX (Gibco; 35050061).

### Cell lines

Cell lines used and generated in this manuscript are detailed in Supplementary Data 1c. RPE-1 TRIM21 KO cells^15^, HEK293T TRIM21 KO cells^79^ and NIH3T3-CAV1-EGFP^78^ were described previously. For stable expression of CAV1-mEGFP and CAV1-mEGFP-Halo, RPE-1 cells were transduced with lentiviral particles at multiplicity ~0.1 transducing units per cell and the GFP-positive population selected by flow cytometry. For stable expression of TRIM21-HA at endogenous levels, RPE-1 TRIM21 KO cells were reconstituted with TRIM21-HA construct under control of the native TRIM21 promoter as described previously^79^.

### Electroporation

Electroporation was performed using the Neon® Transfection System (Thermo Fisher). Cells were washed with PBS and resuspended in Buffer R at a concentration of between 1−8 × 10^7^ cells ml^−1^. For each electroporation reaction 1 − 8 × 10^5^ cells in a 10.5 µl volume were mixed with 2 µl of antibody (typically 0.5 mg/ml) or mRNA (typically 0.5 µM) or protein to be delivered. The mixture was taken up into a 10 µl Neon® Pipette Tip, electroporated at 1400 V, 20 ms, 2 pulses and transferred to media without antibiotics.

### Measurement of fluorescence in live cells

To quantify GFP fluorescence in live cells, images were acquired and analyzed using the IncuCyte live cell analysis system (Sartorius). Within the IncuCyte software, the integrated density (the product of the area and mean intensity) for GFP fluorescence was normalized to total cell area (phase) for each image. Values were normalized to internal controls within each experiment.

### Antibodies

Antibodies and concentrations used for traditional immunoblotting (IB), capillary-based immunoblotting (Jess) and electroporation (EP) are detailed in Supplementary Data 1d. All antibodies used for electroporation were either purchased in azide-free formats or passed through Amicon Ultra-0.5 100 KDa centrifugal filter devices (Millipore) to remove traces of azide and replace buffer with PBS.

### Adv5 neutralization assay

Adenovirus serotype 5 2.6-del CMV-eGFP (Adv5-GFP, Viraquest) was diluted to 1.1 × 10^9^ T.U./mL in PBS, and 16 uL was incubated 1:1 with the anti-hexon reconmbinant humanized IgG1 9C12 or 9C12^H433A^^80^ at indicated concentrations, or PBS. After 1 h incubation at room temperature, complexes were diluted with 250 µL Fluorobrite media and used for Adv5 neutralization assays. For infections, 4 × 10^6^ HEK293T TRIM21 KO cells were electroporated with PBS or R-R-PS ± N^α^-acetylation and resuspended in 2 mL Fluorobrite media. 50 µL of each cell suspension was combined 1:1 with Adv5:9C12 or Adv5:9C12^H433A^ complexes (for immediate infections) or Fluorobrite (for delayed infections) in 96-well plates. For delayed infections, electroporated cells were allowed to adhere to the plate for 2 h, then media was replaced with 50 µL of Fluorobrite and infected with 50 µL Ad5:9C12 complexes. Infection levels were quantified using the IncuCyte system by measuring GFP fluorescence area relative to total cell area 16 h post-infection. Infection levels are plotted relative to Adv5-GFP infection the absence of 9C12 antibody.

### NFκB signalling assay

HEK293T TRIM21 KO cells were transfected with 2 ug pGL4.32 NF-κB luciferase plasmid (Promega), using 12 uL of Viafect (Promega) in 200 uL OptiMEM (Thermo Fisher). Twenty-four hours later 4 × 10^6^ transfected cells were electroporated with PBS or R-R-PS ± N^α^-acetylation and resuspended in 1 mL DMEM media. For infections, Adv5:9C12 complexes were prepared as described above, except Ad5-GFP was diluted to 1.1 × 10^10^, 9C12 was used at 20 ug/mL, and the complex was diluted into 150 µL DMEM after 1 h incubation. 50 µL of the electroporated cell suspension was mixed 1:1 with Adv5:9C12 complexes or PBS (control), then lysed 4 h later in 100 uL of SteadyLite Plus luciferase reporter (PerkinElmer). As an internal control, TNF-α was used at 10 ng/uL. Luminance was recorded on a PheraStar FS (BMG LabTech).

### Immunoblotting

Samples were run on NuPAGE 4–12% Bis-Tris gels (ThermoFisher) and transferred onto nitrocellulose membrane. Membranes were incubated in blocking buffer (PBS, 0.1% Tween20, 5% milk) for 1 h at room temp prior to incubation with antibodies. Antibodies and dilutions (in blocking buffer) used for immunoblotting (IB) are detailed in Supplementary Data 1d. HRP-coupled antibodies were detected by enhanced chemiluminescence (Amersham, GE Healthcare) and X-ray films. IRDye-coupled antibodies were detected using LI-COR Odyssey CLx imaging system. Immunoblot images were cropped in Fiji/ImageJ.

### Capillary-based immunoblotting

RIPA buffer protein extracts were diluted 1:2 in 0.1× sample buffer (bio-techne; 042-195) and run on the Jess Simple Western system using a 12-230 kDa separation module (bio-techne) according to manufacturer’s instructions. Antibodies and dilutions used for capillary-based immunoblotting (Jess) are detailed in Supplementary Data 1d. Protein peak areas were quantified using Compass software (bio-techne) and normalized to internal protein loading controls within each capillary.

### In vitro ubiquitination assays

Ube2W-dependent TRIM21 RING mono-ubiquitination assays were performed in 50 mM Tris pH 7.4, 150 mM NaCl, 2.5 mM MgCl_2_ and 0.5 mM DTT. The reaction components were 2 mM ATP, 1 µM GST-Ube1, 80 µM ubiquitin and the indicated concentrations of Ube2W and TRIM21 RING constructs, respectively. The reaction was stopped by addition of LDS sample buffer containing 50 mM DTT at 4 °C. Next, samples were boiled at 90 °C for 2 min. For reactions using 10 µM TRIM21 RING constructs, visualization was performed by InstantBlue stained LDS-PAGE only. Polyubiquitin chain extension assays were performed as above, but instead of Ube2W, 0.5 µM Ube2N/Ube2V2 were added. For antibody-induced mono-ubiquitination similar conditions were used as for the LDS-PAGE analyzed mono-ubiquitination described above. However, the concentration of TRIM21 RING constructs was reduced to 100 nM and GST-Ube1 to 0.25 µM. Anti-GFP antibody (9F9.F9) was added in one molar equivalent to TRIM21 RING constructs. The reaction was initiated by addition of Ube2W (0, 50, 100, 200 nM). The reaction was stopped by addition of LDS sample buffer at 4 °C. Samples were boiled at 90 °C for 2 min and resolved by LDS-PAGE. TRIM21 RING constructs were visualized using western blot. In vitro reconstitution of Trim-Away ubiquitination events was performed similar to the antibody-induced mono-ubiquitination experiments described above. E2 concentrations were 200 nM Ube2W and 0.5 µM Ube2N/Ube2V2 and His-mEGFP was used as Trim-Away target at 200 nM.

### Acetylation and monoubiquitination assay

N-terminal acetylation of TRIM21 RING constructs and 8xALFAtag-NbGFP^0K^ was catalyzed by the *Chaetomium thermophilum* N-acetyl transferase (NAT) Naa50 catalytic domain residues 82–289 (*Ct*Naa50^82-289^). Acetylation reactions were performed in 50 mM Tris pH 7.4 and 150 mM NaCl for 4 h at 25 °C. The reactions contained 20 µM Substrate, 1 mM Acetyl-CoA and 1 µM *Ct*Naa50. After the Acetylation reaction was finished, it was mixed 1:1 with a Ube2W-ubiquitination mix containing 100 mM Tris pH 7.4, 300 mM NaCl, 5 mM MgCl_2_ and 1 mM DTT, 4 mM ATP, 2 µM GST-Ube1, 160 µM ubiquitin and 2 µM Ube2W. Alternatively, monoubiquitination reactions were performed in 50 mM Tris pH 7.4, 150 mM NaCl, 2.5 mM MgCl_2_ and 0.5 mM DTT. The reaction components were 2 mM ATP, 1 µM GST-Ube1, 80 µM ubiquitin, 5 µM TRIM21 RING constructs and 5 µM 8xALFAtag-NbGFP^0K^. The Ube2W ubiquitinaton reaction was performed for 1 h at 37 °C and stopped by addition of LDS sample buffer containing 50 mM DTT at 4 °C, followed by boiling the samples at 90 °C for 2 min. Visualization was performed by Instant Blue stained LDS-PAGE.

### NMR spectroscopy

Two-dimensional NMR measurements (^15^N-HSQC) were performed at 25 °C on Bruker Avance I 600 MHz spectrometer equipped with 5 mm ^1^H−^13^C−^15^N cryogenic probe. Data was processed with the program Topspin (Bruker BioSpin GmbH, Germany) and analyzed with the program CCPN analysis v2^81^. Samples were buffer exchanged into 50 mM deuterated Tris pH 7.0, 150 mM NaCl and 1 mM deuterated DTT (Cambridge Isotopes, United Kingdom).

Chemical shift perturbations (CSPs) were calculated using the Eq. (1):

1 $${\varDelta}{\delta}_{N,HN}=\sqrt{\left(\right.{\varDelta}{\delta}({\,}^{1}H)^{2}+({\varDelta}{\delta}({\,}^{15}N)^{2} \,*\, 0.14)}$$

where ∆δ_N, HN_ is the CSP, ∆δ(^1^H) and ∆δ(^15^N) are the chemical shift differences between the position of proton or nitrogen signal in absence and presence of titrant. TRIM21 assignments were used from a previous publication^14^.

### Mass spectrometry

Excised protein gel pieces were destained with 50% v/v acetonitrile: 50 mM ammonium bicarbonate. After reduction with 10 mM DTT and alkylation with 55 mM iodoacetamide, the proteins were digested overnight at 37 °C with 6 ng μL^−1^ of Asp-N (Promega, UK). Peptides were extracted in 2% v/v formic acid: 2% v/v acetonitrile and subsequently analyzed by nano-scale capillary LC-MS/MS with an Ultimate U3000 HPLC (Thermo Scientific Dionex, San Jose, USA) set to a flowrate of 300 nL min^−1^. Peptides were trapped on a C18 Acclaim PepMap100 5 μm, 100 μm × 20 mm nanoViper (Thermo Scientific Dionex, San Jose, USA) prior to separation on a C18 T3 1.8 μm, 75 μm × 250 mm nanoEase column (Waters, Manchester, UK). A gradient of acetonitrile eluted the peptides, and the analytical column outlet was directly interfaced using a nano-flow electrospray ionization source, with a quadrupole Orbitrap mass spectrometer (Q-Exactive HFX, Thermo Scientific, USA). For data-dependent analysis a resolution of 60,000 for the full MS spectrum was used, followed by twelve MS/MS. MS spectra were collected over a *m*/*z* range of 300–1800. The resultant LC-MS/MS spectra were searched against a protein database (UniProt KB) using the Mascot search engine program. Database search parameters were restricted to a precursor ion tolerance of 5 ppm with a fragmented ion tolerance of 0.1 Da. Multiple modifications were set in the search parameters: two missed enzyme cleavages, variable modifications for methionine oxidation, cysteine carbamidomethylation, pyroglutamic acid and protein N-term acetylation. The proteomics software Scaffold 4 was used to visualize the fragmented spectra.

### Statistical analysis

Average (mean), standard deviation (s.d.), standard error of the mean (s.e.m) and statistical significance based on Student’s *t* test (two-tailed) and one- or two-way ANOVA were calculated in Microsoft Excel or Graphpad Prism. Significance are sometimes represented with labels ns (not significant, *P* > 0.05), * (*P* ≤ 0.05), ** (*P* ≤ 0.01), *** (*P* ≤ 0.001), **** (*P* ≤ 0.0001).

### Crystallography

Crystals of TRIM21-RING:Ube2W^V30K/D67K/C91K^ complex were grown in 2 nl drops at 10 mg/ml at 17 °C in 0.1 M Bicine pH 9.0, 5% PEG 6000, 0.1 M TCEP hydrochloride. Diffraction experiments were performed at the European Synchrotron Radiation Facility at beamline ID23 using a Dectris PILATUS 6 M detector at a wavelength of 0.984004 Å. The diffraction data at 2.25 Å was processed using XDS. The structure was solved by molecular replacement using Phaser^82^ with TRIM21 RING domain (5OLM^14^) and Ube2W residues 1-118 (2MT6^55^) as search models. Model building and real-space-refinement were carried out in coot^83^, and refinement was performed using REFMAC5 and phenix-refine^84^. Structure visualization and figure generation was carried out in Pymol. For full data collection and refinement statistics see Supplementary Table 1. Model and structure factors have been deposited at the PDB with the accession code 8A58.

### Reporting summary

Further information on research design is available in the Nature Portfolio Reporting Summary linked to this article.

## Supplementary information

Supplementary Information

Description of Additional Supplementary Files

Supplementary Data 1

Reporting Summary

## Data Availability

Source data are provided within this paper. The TRIM21 RING:Ube2W crystal structure with model and structure factors have been deposited at the PDB with the accession code 8A58. Previously published TRIM21 RING crystal structure data used in this study are 5OLM, 6S53 and 7BBD. All other data are available from the corresponding authors on request. Source data are provided with this paper.

## Source data

Source Data
