# Supplementary material for: Trim-Away ubiquitinates and degrades lysine-less and N-terminally acetylated substrates
Source: Nat Commun. 2023 Apr 15;14:2160. doi: 10.1038/s41467-023-37504-x (PMC10105713; doi:10.1038/s41467-023-37504-x)
Supplement: Supplementary file 1 — Supplementary Information [file 41467_2023_37504_MOESM1_ESM.pdf]

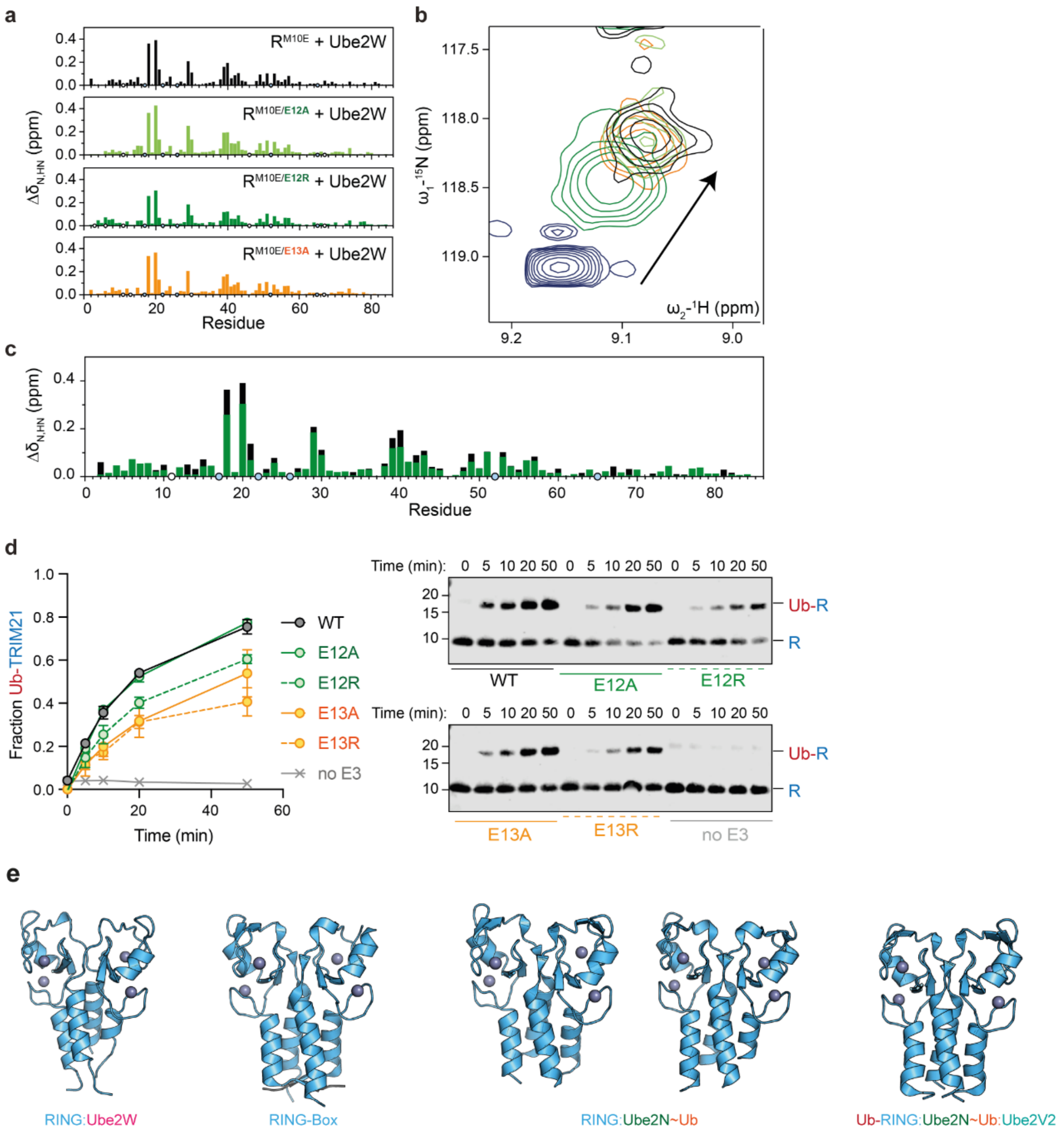

Supplementary Fig. 1: Interaction of Ube2W with TRIM21 RING. a Histograms of chemical shift perturbations (CSP) shown against the sequence of TRIM21 RING^{M10E} (R^{M10E}). These CSPs result from NMR titrations of Ube2W$^{V30K/D67K/C91K}$ against ^{15}N-labelled TRIM21 tri-ionic mutants at a 1:1 molar ratio. Blue circles indicate proline residues, white circles missing assignments. **b** A part of ^{15}N-HSQC spectral overlay of R^{M10E} in absence (blue) or presence of 1:1 molar equivalent of Ube2W$^{V30K/D67/C91K}$. In addition, spectra of TRIM21 mutants (E12A in light green, E12R in dark green and E13A in orange) are shown in presence of 1:1 molar equivalent of Ube2W$^{V30K/D67/C91K}$. **c** Histograms shown in (A) are here shown as an overlay. **d** Ube2W-mediated TRIM21 RING monoubiquitination assay. Shown is a time-course, where error bars represent s.e.m. from n = 3 independent experiments. Western blots are representative of all replicates. **e** Shown are RING dimers of different TRIM21 complexes RING:Ube2W (8A58 [http://doi.org/10.2210/pdb8A58/pdb]), RING-Box (5OLM [http://doi.org/10.2210/pdb5OLM/pdb])[14], RING:Ube2N~Ub (two RING dimers in asymmetric unit; 6S53 [http://doi.org/10.2210/pdb6S53/pdb])[30], Ub-RING:Ube2N~Ub:Ube2V2 (7BBD [http://doi.org/10.2210/pdb7BBD/pdb])[38]. Zn^{2+}-atoms are shown as grey spheres, the isopeptide bond is marked by an arrow and polar interactions are indicated by dashed black lines.

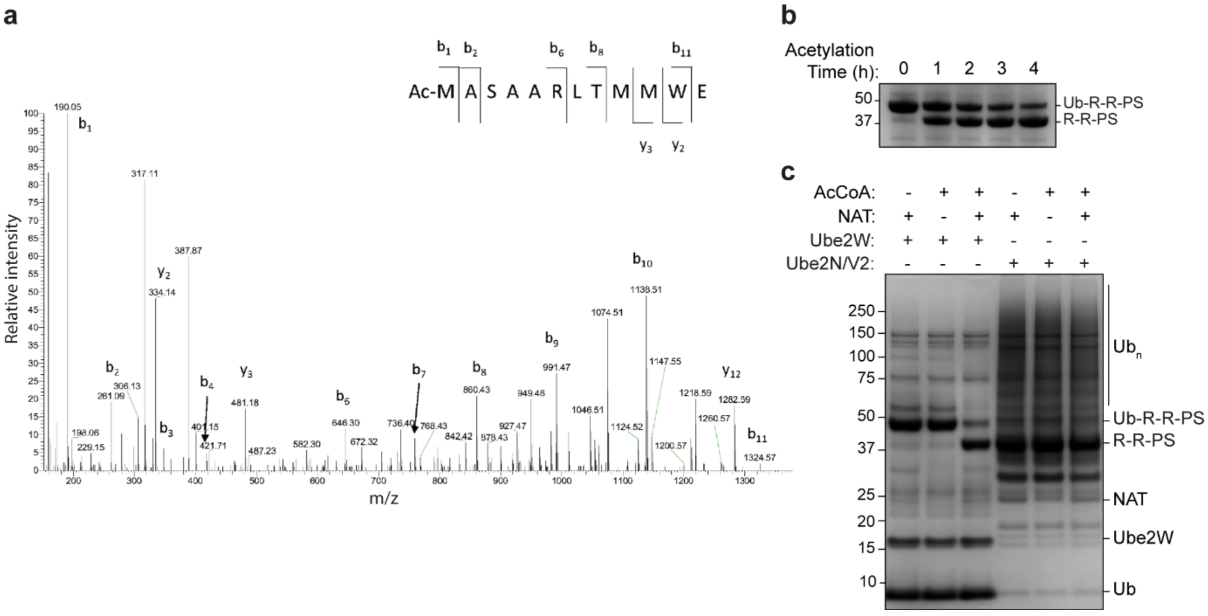

Supplementary Fig. 2: N-terminal ubiquitination of TRIM21 RING. a LC-MS/MS spectra of R-R-PS after 4 h acetylation reaction show N-terminally acetylated TRIM21 N-terminal peptides after digestion with the protease N-Asp. **b** Instant-Blue-stained gels showing Ube2W-mediated mono-ubiquitination of R-R-PS. Before the ubiquitination reaction, acetylation reactions were performed for the indicated times. Representative example from 2 independent experiments. **c** Ubiquitination reaction upon incubation with Ube2W or Ube2N/Ube2V2 for 1 h after 4 h of R-R-PS N-terminal acetylation. N-terminal acetylation does not impact ubiquitin smearing characteristic of free K63-chain formation. Representative example from 2 independent experiments.

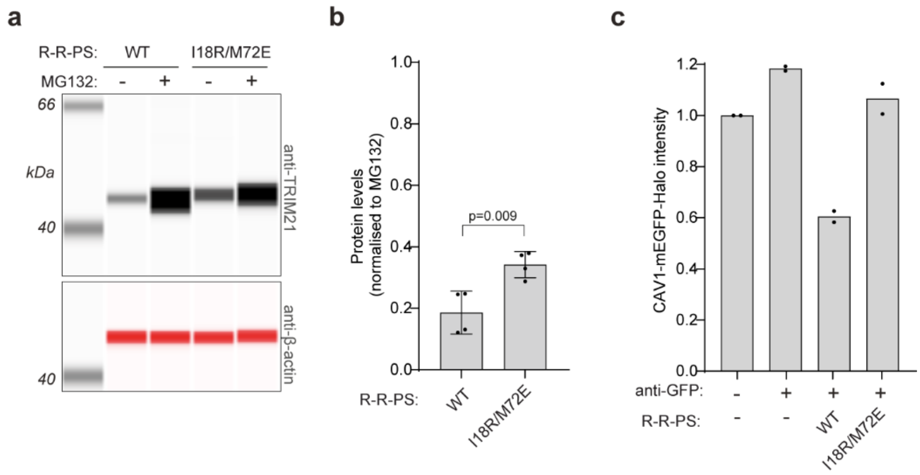

Supplementary Fig. 3: RING-inactivating mutations affect ligase turnover and substrate degradation. a,b R-R-PS proteins containing WT or I18R/M72E mutant RING domains were electroporated into RPE-1 TRIM21 KO cells ± MG132 and whole cell lysates harvested 1 hour later for immunoblotting. Lane view (**a**) and quantification (**b**) of R-R-PS protein levels normalized to MG132 condition. **c** RPE-1 TRIM21 KO cells expressing CAV1-mEGFP-Halo were electroporated with PBS or anti-GFP antibody ± the indicated R-R-PS proteins and fluorescence measured 3 hours later using the IncuCyte system. Graph shows mean and s.e.m. (**b**) or mean (**c**) from 4 (**b**) or 2 (**c**) independent experiments (black dots). Statistical significance based on two-tailed Student's *t*-test. Source data are provided as a Source Data file.

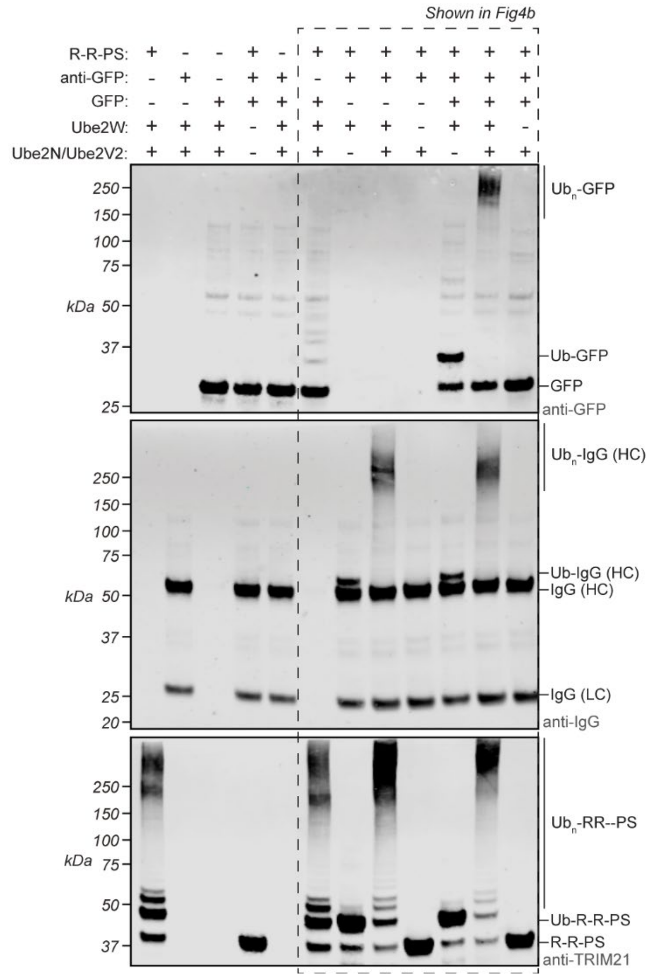

Supplementary Fig. 4: TRIM21 independently ubiquitinates itself and substrate. Western blots of in vitro ubiquitination reactions using combinations of R-R-PS, anti-GFP antibody, GFP, Ube2W and Ube2N/Ube2V2. Representative example from 2 independent experiments. Dashed line outlines region shown in Fig. 4b.

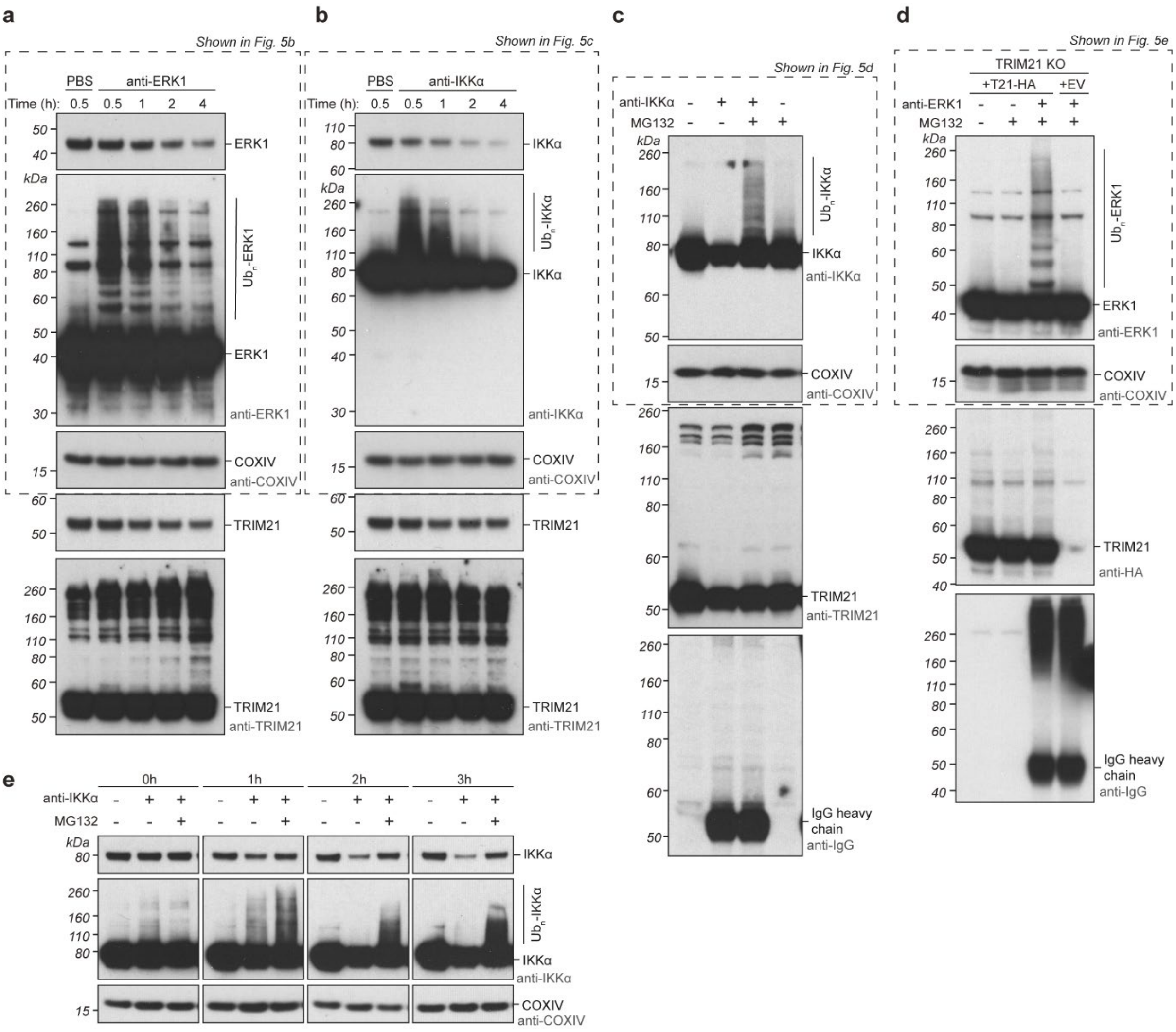

Supplementary Fig. 5: Substrate ubiquitination during Trim-Away in live cells. a-d Western blots of TRIM21 and antibody from Trim-Away experiments shown in Fig. 5b-e. Dashed lines outline regions shown in Fig. 5b-e. **e** Western blot of Trim-Away time-course experiment using anti-IKKα antibody in the presence or absence of MG132. Proteasome inhibition rescues IKKα degradation and leads to the accumulation of ubiquitinated protein. Representative example from 2 independent experiments.

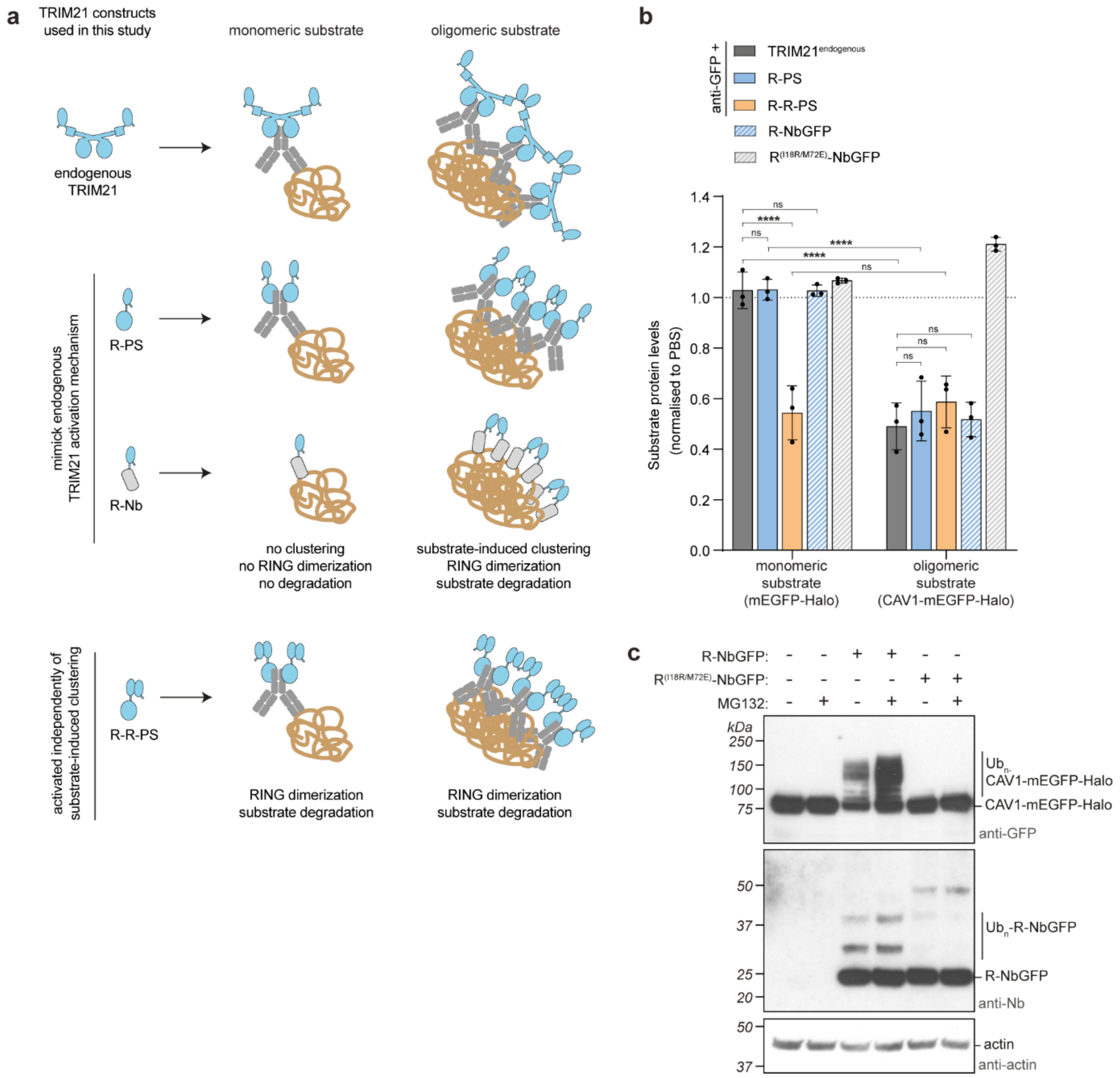

Supplementary Fig. 6: RING-nanobody constructs mimic endogenous TRIM21. a Schematic of TRIM21 constructs used in this study and respective requirements for substrate-induced clustering for activation. **b** Experimental validation of schematic shown in (**a**). RPE-1 cells expressing either monomeric (mEGFP-Halo) or oligomeric (CAV1-mEGFP-Halo) substrates were electroporated with anti-GFP antibody plus TRIM21 RING-PRYSPRY constructs (solid colours) or TRIM21 RING-NbGFP constructs alone (striped colours) and substrate fluorescence measured 6 hours later using the IncuCyte system. Graph shows mean and s.e.m. from n = 3 independent experiments (black dots). Statistical significance based on two-way ANOVA and represented with ns (not significant, P>0.05), *** (P≤0.001), **** (P≤0.0001). **c** Western blot of Trim-Away experiment using WT R-NbGFP or a mutant incapable of catalysing ubiquitination ($R^{I18R/M72E}$-NbGFP). Representative example from 3 independent experiments. Source data are provided as a Source Data file.

Supplementary Figure 7

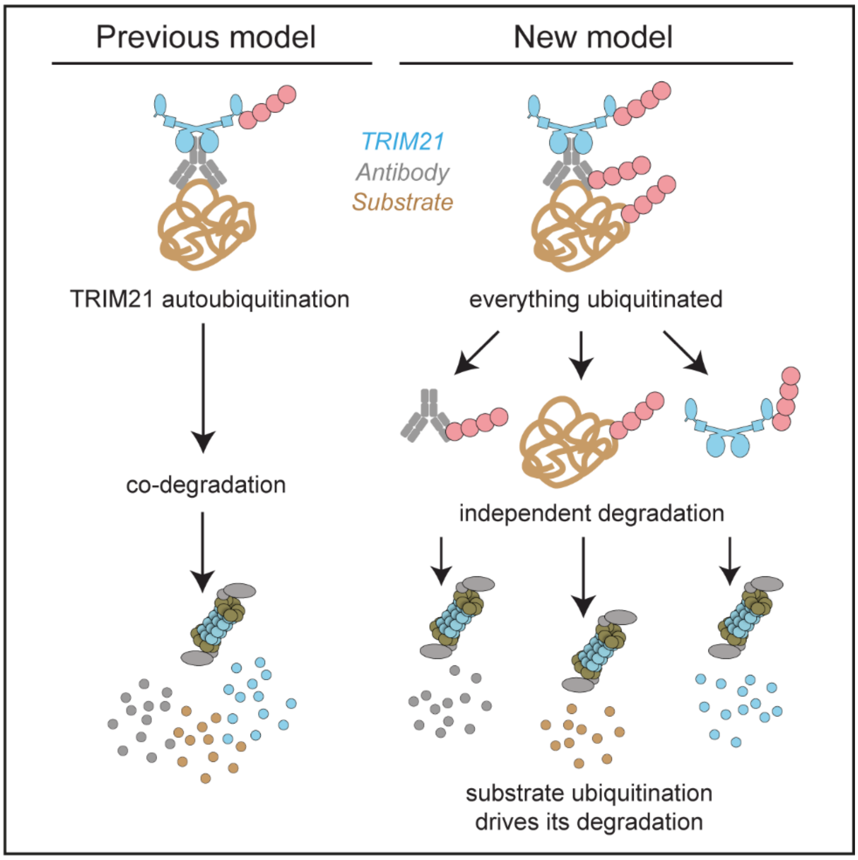

Supplementary Fig. 7: New model for substrate degradation by Trim-Away. See discussion for details.

	T21-R:Ube2W
**Data collection**	
Space group	P121
Cell dimensions	
a, *b*, *c* (Å)	62.82, 75.482, 63.835
α, β, γ (°)	90.0, 119.307, 90.0
Resolution (Å)	29.46-2.25 (2.33-2.25)
R_{meas}	4.8 (65.9)
$CC_{1/2}$ (%)	99.9 (85.9)
$I / \sigma I$	16.49 (2.01)
Completeness (%)	95.5 (96.3)
Redundancy	3.3 (3.6)
**Refinement**	
Resolution (Å)	29.46-2.25 (2.33-2.25)
No. reflections	23634 (2355)
R_{work} / R_{free}	0.22/0.26 (0.36/0.42)
No. atoms	3515
Protein	3476
Ligand/ion	4
Water	35
B-factors	
Protein	83.06
Ligand/ion	56.95
Water	78.61
R.m.s. deviations	
Bond lengths (Å)	0.011
Bond angles (°)	1.41

*Values in parentheses are for highest-resolution shell.

Supplementary Table 1: Crystal structure data collection and refinement statistics. The TRIM21 RING:Ube2W crystal structure with model and structure factors have been deposited at the PDB with the accession code 8A58 [http://doi.org/10.2210/pdb8A58/pdb]. Statistics for both data integration and model refinement are given.

SOURCE DATA

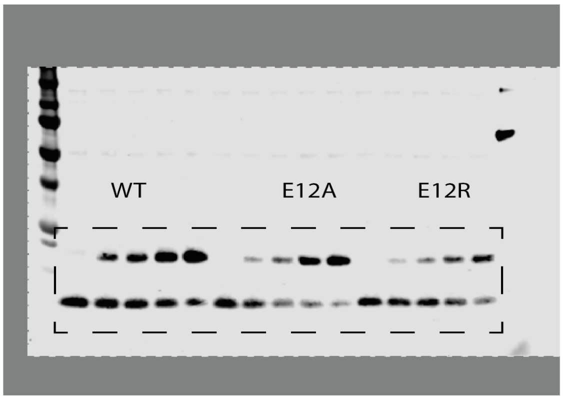

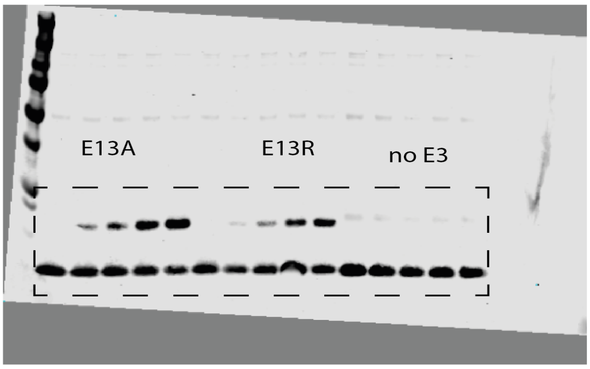

Uncropped blots from Supplementary Fig. 1d

b

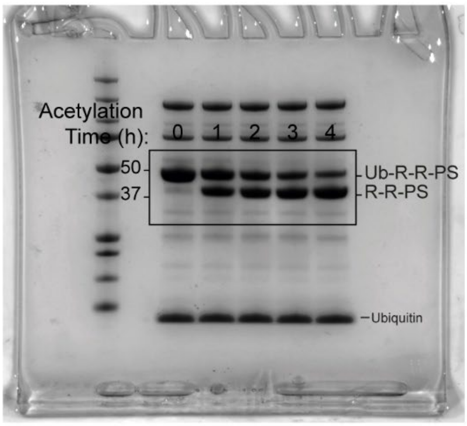

c

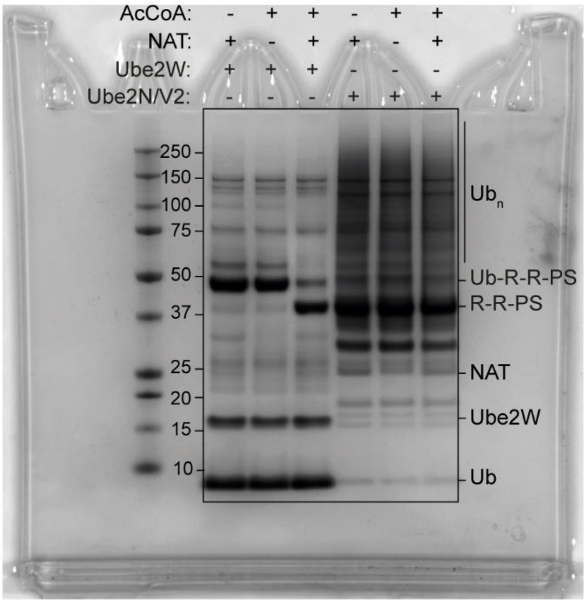

Uncropped gels from Supplementary Fig. 2b,c

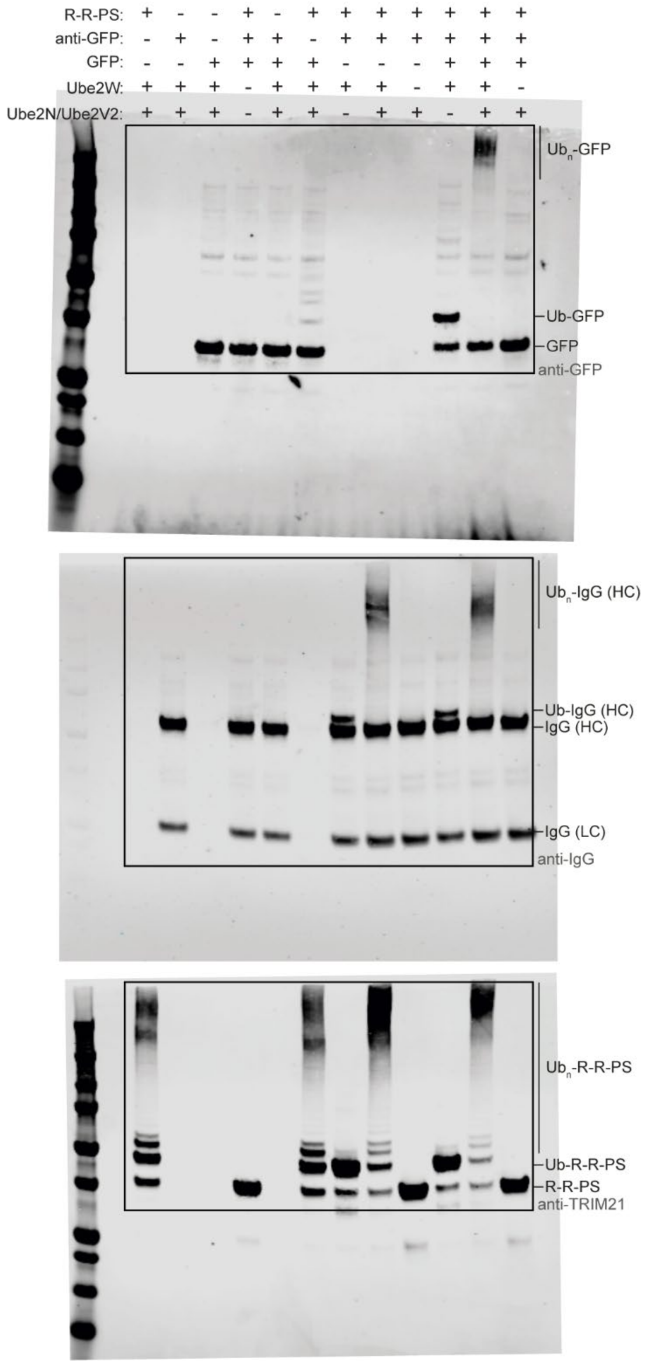

Uncropped blots from Supplementary Fig. 4

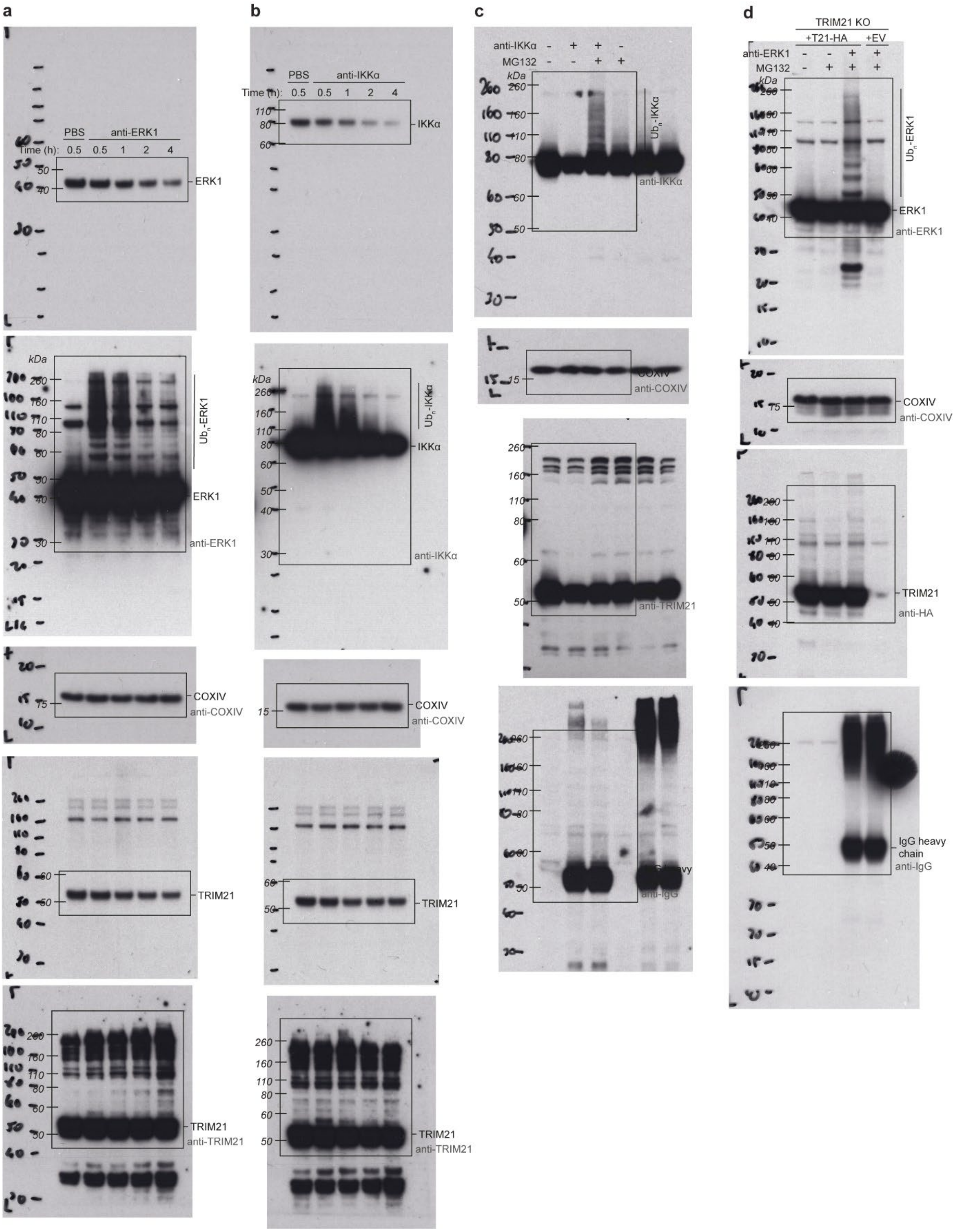

Uncropped blots from Supplementary Fig. 5a-d

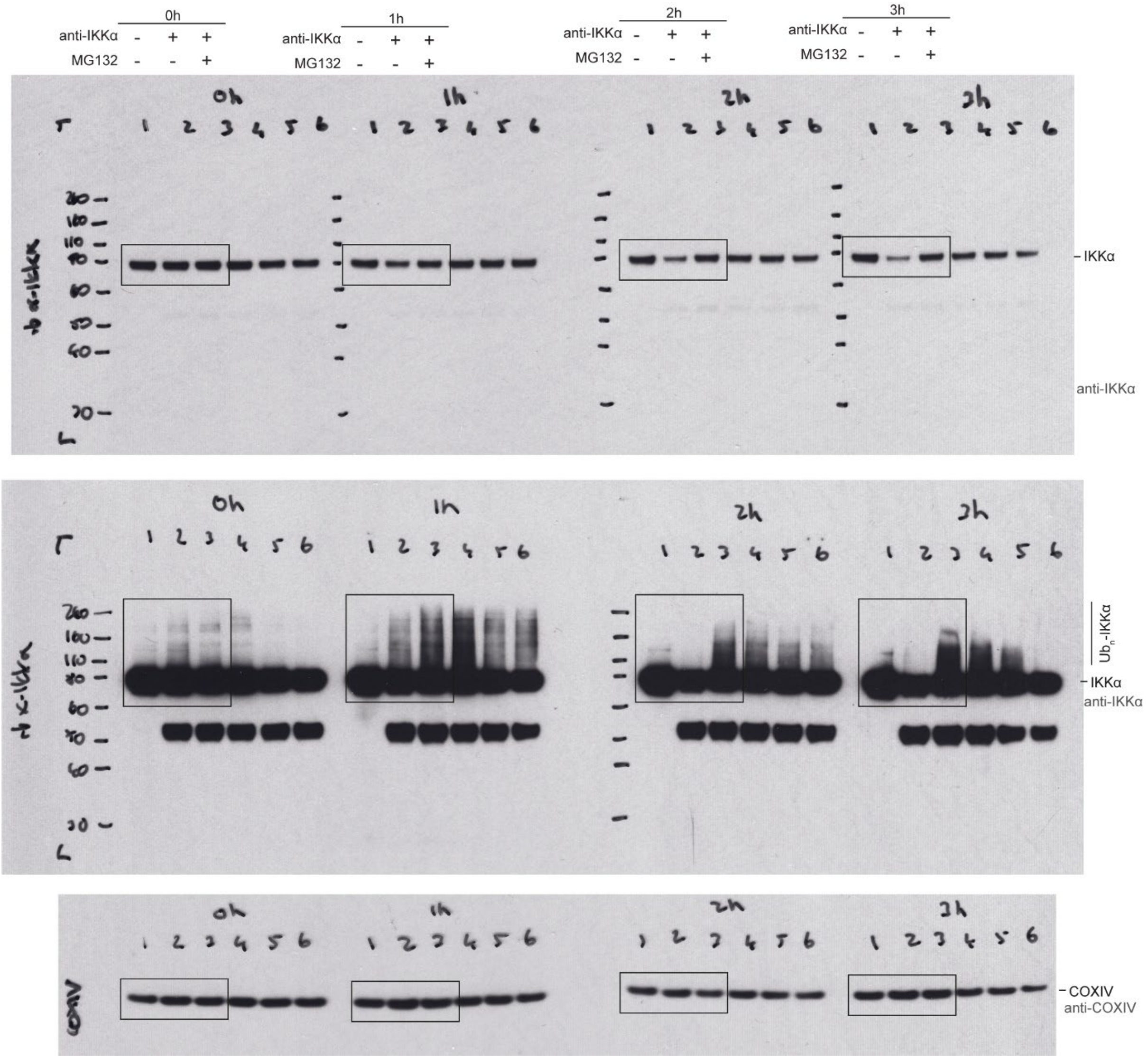

Uncropped blots from Supplementary Fig. 5e

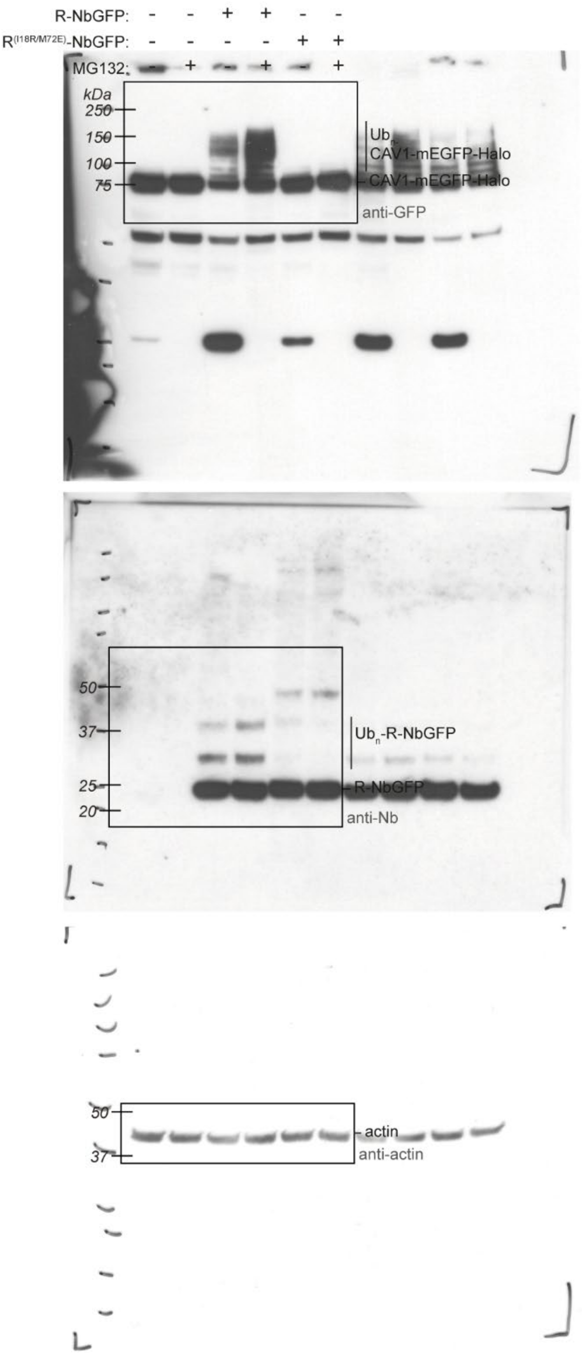

Uncropped blots from Supplementary Fig. 6c
