# Supplementary material for: Trim-Away ubiquitinates and degrades lysine-less and N-terminally acetylated substrates
Source: Nat Commun. 2023 Apr 15;14:2160. doi: 10.1038/s41467-023-37504-x (PMC10105713; doi:10.1038/s41467-023-37504-x)
Supplement: Supplementary file 2 — Description of Additional Supplementary Files [file 41467_2023_37504_MOESM2_ESM.docx]

File Name: Supplementary Information

Description: Supplementary Figures 1-7 and Supplementary Table 1.

File Name: Supplementary Data 1

Description: Lists of plasmids (**a**), purified proteins (**b**), cell lines (**c**), antibodies (**d**) and commercial assays/software (**e**) used in this study.

File Name: Source Data

Description: Raw data corresponding to plotted graphs within main and supplementary figures. Uncropped gels and blots from main figures.
