# Supplementary material for: Trim-Away ubiquitinates and degrades lysine-less and N-terminally acetylated substrates
Source: Nat Commun. 2023 Apr 15;14:2160. doi: 10.1038/s41467-023-37504-x (PMC10105713; doi:10.1038/s41467-023-37504-x)
Supplement: Supplementary file 4 — Reporting Summary [file 41467_2023_37504_MOESM4_ESM.pdf]

nature portfolio

Corresponding author(s): James, LC

Last updated by author(s): Mar 10, 2023

Reporting Summary

Nature Portfolio wishes to improve the reproducibility of the work that we publish. This form provides structure for consistency and transparency in reporting. For further information on Nature Portfolio policies, see our Editorial Policies and the Editorial Policy Checklist.

Statistics

For all statistical analyses, confirm that the following items are present in the figure legend, table legend, main text, or Methods section.

n/a	Confirmed	
☐	☒	The exact sample size (*n*) for each experimental group/condition, given as a discrete number and unit of measurement
☐	☒	A statement on whether measurements were taken from distinct samples or whether the same sample was measured repeatedly
☐	☒	The statistical test(s) used AND whether they are one- or two-sided<br>*Only common tests should be described solely by name; describe more complex techniques in the Methods section.*
☒	☐	A description of all covariates tested
☐	☒	A description of any assumptions or corrections, such as tests of normality and adjustment for multiple comparisons
☐	☒	A full description of the statistical parameters including central tendency (e.g. means) or other basic estimates (e.g. regression coefficient) AND variation (e.g. standard deviation) or associated estimates of uncertainty (e.g. confidence intervals)
☐	☒	For null hypothesis testing, the test statistic (e.g. *F*, *t*, *r*) with confidence intervals, effect sizes, degrees of freedom and *P* value noted<br>*Give P values as exact values whenever suitable.*
☒	☐	For Bayesian analysis, information on the choice of priors and Markov chain Monte Carlo settings
☒	☐	For hierarchical and complex designs, identification of the appropriate level for tests and full reporting of outcomes
☒	☐	Estimates of effect sizes (e.g. Cohen's *d*, Pearson's *r*), indicating how they were calculated

Our web collection on statistics for biologists contains articles on many of the points above.

Software and code

Policy information about availability of computer code

Data collection	Crysallographic data collection was performed using the setup provided by the European Synchrotron Radiation Facility at beamline ID23
Data analysis	Microsoft Excel (version office 2019)<br>GraphPad Prism (version 9.5.1)<br>IncuCyte Software, Sartorius (version 2021C)<br>Compass software for simple western, Bio-Techne (version 5.0.1)<br>Fiji/ImageJ (version 2.3.0/1.54c)<br>PyMOL (version 1.8.2.3)<br>Coot (versions 0.8.9 and 0.9)<br>XDS (version 31 January 2019)<br>Refmac5 (version 5.5)<br>Phenix-Refine (version 1.18.2-3874)<br>Phaser (version 2.8)

For manuscripts utilizing custom algorithms or software that are central to the research but not yet described in published literature, software must be made available to editors and reviewers. We strongly encourage code deposition in a community repository (e.g. GitHub). See the Nature Portfolio guidelines for submitting code & software for further information.

Data

Policy information about availability of data

All manuscripts must include a data availability statement. This statement should provide the following information, where applicable:

- Accession codes, unique identifiers, or web links for publicly available datasets
- A description of any restrictions on data availability
- For clinical datasets or third party data, please ensure that the statement adheres to our policy

Source data are provided within this paper. The crystal structure with model and structure factors have been deposited at the PDB with the accession code 8A58 (10.2210/pdb8A58/pdb). All other data are available from the corresponding authors on reasonable request.

Human research participants

Policy information about studies involving human research participants and Sex and Gender in Research.

Reporting on sex and gender	n.a.
Population characteristics	n.a.
Recruitment	n.a.
Ethics oversight	n.a.

Note that full information on the approval of the study protocol must also be provided in the manuscript.

Field-specific reporting

Please select the one below that is the best fit for your research. If you are not sure, read the appropriate sections before making your selection.

☒ Life sciences ☐ Behavioural & social sciences ☐ Ecological, evolutionary & environmental sciences

For a reference copy of the document with all sections, see nature.com/documents/nr-reporting-summary-flat.pdf

Life sciences study design

All studies must disclose on these points even when the disclosure is negative.

Sample size	Sample size taken as number of repeats of each experiment and always more than or equal to n=2.
Data exclusions	No data were excluded from any analysis
Replication	All experiments were independently replicated at least twice. All attempts at replication were successful
Randomization	n.a.
Blinding	n.a.

Reporting for specific materials, systems and methods

We require information from authors about some types of materials, experimental systems and methods used in many studies. Here, indicate whether each material, system or method listed is relevant to your study. If you are not sure if a list item applies to your research, read the appropriate section before selecting a response.

Materials & experimental systems

n/a	Involved in the study	
☐	☒	Antibodies
☐	☒	Eukaryotic cell lines
☒	☐	Palaeontology and archaeology
☒	☐	Animals and other organisms
☒	☐	Clinical data
☒	☐	Dual use research of concern

Methods

n/a	Involved in the study	
☒	☐	ChIP-seq
☒	☐	Flow cytometry
☒	☐	MRI-based neuroimaging

Antibodies

Antibodies used

humanised anti-hexon IgG1 9C12
humanised anti-hexon IgG1 9C12H433A
mouse anti-GFP clone 9F9.F9
mouse anti-GFP (clones 7.1 and 13.1)
NbALFA-msIgG1-Fc
NbALFA-HRP
rabbit anti-ERK1 clone Y72
rabbit anti-IKKα clone Y463
rabbit anti-TRIM21 clone D1O1D
mouse anti-TRIM21 clone D-12
rabbit anti-vhh
rat anti-HA clone 3F10 HRP-conjugated
rabbit anti-Vinculin clone EPR8185
rabbit anti-COXIV
rabbit anti-Hsp60
Rabbit anti-Mouse IgG HRP-conjugated
Goat anti-Mouse light chain specific, HRP-conjugated
Goat anti-Mouse IgG, IRDye 800CW
Goat anti-Rabbit IgG HRP-conjugated
Mouse anti-Rabbit light chain specific, HRP-conjugated
Goat anti-Rabbit IgG, IRDye 680RD

Validation

Each antibody has been validated by the vendor:

https://doi.org/10.4049/jimmunol.1502601
https://doi.org/10.4049/jimmunol.1502601
http://antibodyregistry.org/AB_218216
http://antibodyregistry.org/AB_390913
NanoTag; N1582
NanoTag; N1501-HRP
http://antibodyregistry.org/AB_732202
http://antibodyregistry.org/AB_733070
http://antibodyregistry.org/AB_2800177
http://antibodyregistry.org/AB_628286
http://antibodyregistry.org/AB_2734123
http://antibodyregistry.org/AB_390917
http://antibodyregistry.org/AB_11144129
http://antibodyregistry.org/AB_2783000
http://antibodyregistry.org/AB_2118931
http://antibodyregistry.org/AB_2636929
http://antibodyregistry.org/AB_805324
http://antibodyregistry.org/AB_2687825
http://antibodyregistry.org/AB_228338
http://antibodyregistry.org/AB_827270
http://antibodyregistry.org/AB_2721181

Eukaryotic cell lines

Policy information about cell lines and Sex and Gender in Research

Cell line source(s)

HEK293T (ATCC; CRL-3216)
HEK293T TRIM21 KO (https://doi.org/10.7554/eLife.32660)

	hTERT-RPE-1 (ATCC; CRL-4000)<br>hTERT-RPE-1 TRIM21 KO (https://doi.org/10.1038/s41594-021-00560-2)<br>hTERT-RPE-1 TRIM21 KO TRIM21-HA (This paper)<br>hTERT-RPE-1 CAV1-mEGFP (This paper)<br>hTERT-RPE-1 CAV1-mEGFP-Halo (This paper)<br>hTERT-RPE-1 mEGFP-Halo (This paper)<br>hTERT-RPE-1 TRIM21 KO CAV1-mEGFP-Halo (This paper)<br>NIH3T3-CAV1-EGFP (https://doi.org/10.1038/ncomms7867)
Authentication	HEK293T (ATCC; CRL-3216) - Authentication: Morphology<br>HEK293T TRIM21 KO (https://doi.org/10.7554/eLife.32660) - Authentication: Morphology, TRIM21 western blot<br>hTERT-RPE-1 (ATCC; CRL-4000) - Authentication: Morphology<br>hTERT-RPE-1 TRIM21 KO (https://doi.org/10.1038/s41594-021-00560-2) - Authentication: Morphology, TRIM21 western blot<br>hTERT-RPE-1 TRIM21 KO TRIM21-HA (This paper) - Authentication: Morphology, TRIM21 western blot<br>hTERT-RPE-1 CAV1-mEGFP (This paper) - Authentication: Morphology, fluorescence microscopy, GFP western blot<br>hTERT-RPE-1 CAV1-mEGFP-Halo (This paper) - Authentication: Morphology, fluorescence microscopy, GFP western blot<br>hTERT-RPE-1 mEGFP-Halo (This paper) - Authentication: Morphology, fluorescence microscopy, GFP western blot<br>hTERT-RPE-1 TRIM21 KO CAV1-mEGFP-Halo (This paper) - Authentication: Morphology, fluorescence microscopy, TRIM21 and GFP western blot<br>NIH3T3-CAV1-EGFP (https://doi.org/10.1038/ncomms7867) - Authentication: Morphology, fluorescence microscopy, GFP western blot
Mycoplasma contamination	All cell lines were routinely screened and determined to be mycoplasma-free
Commonly misidentified lines<br>(See ICLAC register)	No commonly misidentified cell lines were used in this study
